# Corrosion inhibition mechanisms of 2-mercaptobenzothiazole on AA2024 T3 aluminium alloy

**DOI:** 10.1038/s41529-025-00653-z

**Published:** 2025-08-03

**Authors:** Vishant Garg, Maxime Magnan, Sandrine Zanna, Antoine Seyeux, Frédéric Wiame, Vincent Maurice, Philippe Marcus

**Affiliations:** 1 PSL University, CNRS—Chimie ParisTech, Institut de Recherche de Chimie Paris, Physical Chemistry of Surfaces Research Group, Paris, France; 2 Present Address: Institute of Research and Technology—Materials, Metallurgy, and Processes (IRT-M2P), Duppigheim, France

**Keywords:** Corrosion, Characterization and analytical techniques, Surface spectroscopy

## Abstract

The mechanisms of 2-mercaptobenzothiazole (2-MBT) adsorption and corrosion inhibition on the aerospace AA2024 T3 aluminium alloy have been investigated using electrochemistry and advanced surface analyses. Electrochemical methods were used to measure the degree of corrosion protection in neutral chloride media, while advanced surface analysis techniques were employed to determine the interfacial interaction and inhibitor action mechanisms. It is shown that 2-MBT effectively inhibits corrosion of the alloy, reducing its susceptibility to corrosion attack and its corrosion rate. Surface analysis, including the use of ToF-SIMS 3-D chemical mapping, confirms 2-MBT adsorption on partially dealloyed intermetallic particles (IMPs) along with the presence of a thin 2-MBT layer on the top-most alloy surface. Chloride ions breakdown the native oxide film, allowing 2-MBT to adsorb on IMPs, thereby inhibiting further localized corrosion on these particles, while the 2-MBT layer on the surface protects the alloy matrix from surface oxidation.

## Introduction

The AA2024 alloy is a legacy aluminium alloy that has been used in the aerospace industry for several decades^1^. This alloy belongs to the 2xxx series of aluminium alloys where the major alloying element is copper, along with minor alloying elements such as magnesium, iron, manganese, silicon, zinc, and traces of other elements^2^. These alloys are especially known for their high strength, high heat resistance, and good machinability, thereby allowing them to be used in critical components such as engine parts and aerospace components, to name a few^3^. Despite the formation of a few nanometres’ thick passive oxide film on the AA2024 alloy surface, like for other aluminium alloys, it is prone to localised corrosion attack in aggressive environments.

One of the major reasons for corrosion attack on the AA2024 alloy is the presence of secondary phase particles, also known as intermetallic particles (IMPs), and the occurrence of defects in the oxide film near these particles which can facilitate the transfer of the corrosive ions through the oxide film. The most common IMPs found in the AA2024 alloy are the Al_2_CuMg (S phase)^4–9^, Al-Cu-Mn-Fe-(Si)^6–9^, Al_2_Cu (θ phase)^6–9^, and the Mg_2_Si^7–9^ particles. These particles, formed during alloy solidification, are rich in alloying elements, thereby making them either anodic or cathodic with respect to the surrounding aluminium matrix^9–11^. This in turn leads to galvanic activity – both within the particles and between the particles and the matrix, resulting in various forms of corrosion attack such as dealloying of IMPs, trenching around the particles, pitting, intergranular attack, and severe localised corrosion (SLC) attack in the presence of corrosive agents such as halide ions^11–19^.

2-mercaptobenzothiazole, more commonly referred to as 2-MBT, is an organic heterocyclic compound that has been well-documented as an effective corrosion inhibitor for copper^20–27^. The molecule, C_7_H_5_NS_2_, contains two sulfur atoms and one nitrogen atom which act as hetero-atoms and aid in bonding with the metals in both metallic and oxidised states. Previous works have shown that 2-MBT inhibits both corrosion and oxidation of copper in acidic, neutral, and alkaline pH environments^24–26^. However, the corrosion inhibition effectiveness of 2-MBT on copper is much greater when adsorbed on metallic surfaces than oxidised surfaces, even in aggressive acidic chloride environments^24^. While there remains no doubt on its inhibition properties on copper, its use and mechanism for protecting aluminium alloys is still debated.

The use of 2-MBT as a potential corrosion inhibitor for aluminium alloys, and specifically the AA2024 alloy, has been investigated by some researchers in the last few years, mainly utilising electrochemical techniques to qualify the performance and efficiency of this inhibitor^28–33^. Özkan et al.^28^ demonstrated that, among 78 different chemical compounds previously used as corrosion inhibitors, 2-MBT ranks in the top 10 most effective inhibitors for the AA2024 alloy. They also concluded that molecules containing phosphorous as their heteroatoms are the most effective corrosion inhibitors for the AA2024 alloy, followed by sulfur, nitrogen, and finally oxygen, which is the least effective. Meanwhile, a study investigating the effect of inhibitor structure found that 2-MBT is more effective on AA2024 than on AA7075 alloy, which was attributed to the higher concentration of copper in the AA2024 alloy^29^.

Balaskas et al.^30^ and Zheludkevich et al.^31^ also conducted long-term immersion tests along with electrochemical measurements in their works on inhibitors for the AA2024 alloy. They observed that 2-MBT decreases the rate of both the anodic and cathodic reactions that occur during attack. Furthermore, they proposed that the cathodic reactions were inhibited due to the precipitation of 2-MBT on the copper rich intermetallic particles. However, Visser et al.^32^ reported that 2-MBT exhibits a reversible behaviour on the AA2024 alloy, i.e., the protection offered by 2-MBT lapsed once the alloy was removed from the inhibitor-containing solution. Therefore, they concluded that the concentration of 2-MBT needs to be maintained for optimal protection. Although these studies yielded promising results, they were predominantly focused on evaluating inhibitor performances from a variety of inhibitors using electrochemical tests. The mechanisms of 2-MBT molecule adsorption and inhibitor action on the AA2024 alloy have remained elusive until now. Additionally, the relationship between inhibitor adsorption and its reversibility properties, a critical factor in understanding the nature of the inhibitor, has been unexplored.

The adsorption mechanisms of 2-mercaptobenzimidazole (2-MBI), an inhibitor with a similar structure to 2-MBT, on various aluminium alloys have been investigated by Kozlica et al.^34–36^. Even though these studies offer some perspectives towards metal-inhibitor interactions, we have demonstrated in our previous works that the bonding mechanisms of 2-MBI and 2-MBT are quite distinct on the same material (copper) and same environment (pH, electrolyte/ions)^24,37^. Therefore, it is essential to study the 2-MBT – AA2024 system independently to determine the mechanisms of molecule adsorption and inhibitor action on the alloy.

The aim of the present work was to elucidate how 2-MBT adsorbs on the AA2024 T3 aluminium alloy in a near-neutral chloride aqueous solution to facilitate greater insight on inhibitor-alloy systems. While the question is rather straightforward, several sub-questions arise to comprehend this matter. These include: where does 2-MBT adsorb on the alloy surface – the alloy matrix or the intermetallic particles, and is there a preferential adsorption site? Is there any key difference between the adsorption mechanisms on the aluminium alloy matrix and the IMPs? Does the presence of chloride ions assist in 2-MBT adsorption, or does it hinder the process? Finally, while it is known from previous works that 2-MBT inhibits corrosion, does it also restrict oxidation of the alloy? These questions are crucial to unravel the mechanisms of inhibitor bonding and action and addressing them will enhance the understanding of alloy-inhibitor interactions.

To this end, we first investigated the corrosion inhibition properties and effectiveness of 2-MBT on the AA2024 T3 aluminium alloy in a neutral chloride environment, using electrochemical methods such as open circuit potential (OCP) monitoring, linear polarisation resistance (LPR) measurements, and potentiodynamic polarisation (PDP) tests. Next, the adsorption mechanisms and subsequently the inhibition mechanisms were studied using advanced surface analytical techniques. X-ray photoelectron spectroscopy (XPS) and time-of-flight secondary ion mass spectrometry (ToF-SIMS) were used to determine whether the inhibitor molecule effectively adsorbs on the alloy or not. Finally, 3-D chemical mapping using ToF-SIMS was performed to establish the sites of 2-MBT adsorption and the mechanisms of inhibition action for corrosion protection.

## Results and Discussion

### Electrochemical Assessment Of Corrosion Inhibition by 2-MBT

**Fig. 1:**
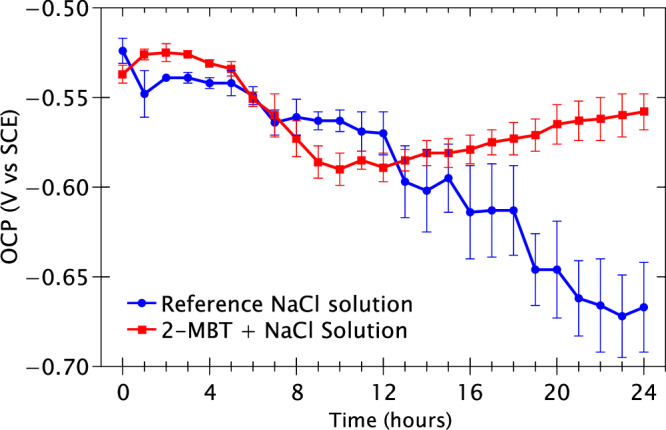
OCP monitoring of the AA2024 T3 samples immersed in the reference NaCl solution and the 2-MBT-containing NaCl solution over 24 h of experimental time.

To evaluate the corrosion inhibition properties of 2-MBT on the AA2024 T3 alloy, electrochemical measurements were performed in the reference solution and the 2-MBT-containing solution. Figure 1 presents the OCP variation as a function of time over 24 h of immersion.

It is observed that for the first 5 h of immersion, the samples immersed in the reference and inhibitor-containing NaCl solutions present rather stable and similar potentials within the range of −0.52 to −0.55 V vs SCE. After 5 h of immersion, the sample immersed in the reference solution exhibits a decreasing trend for OCP till the end of the experiment at 24 h. For the sample immersed in the 2-MBT-containing solution, the OCP decreases between 5 and 10 h of immersion followed by an increasing trend from 10 h of immersion to the end of the experiment. The final OCP values after 24 h of immersion were −0.67 ± 0.03 V and −0.56 ± 0.01 V vs SCE for the samples in the reference and 2-MBT-containing solutions, respectively. This result indicates that 2-MBT inhibits corrosive attack on the AA2024 alloy in these conditions.

**Fig. 2:**
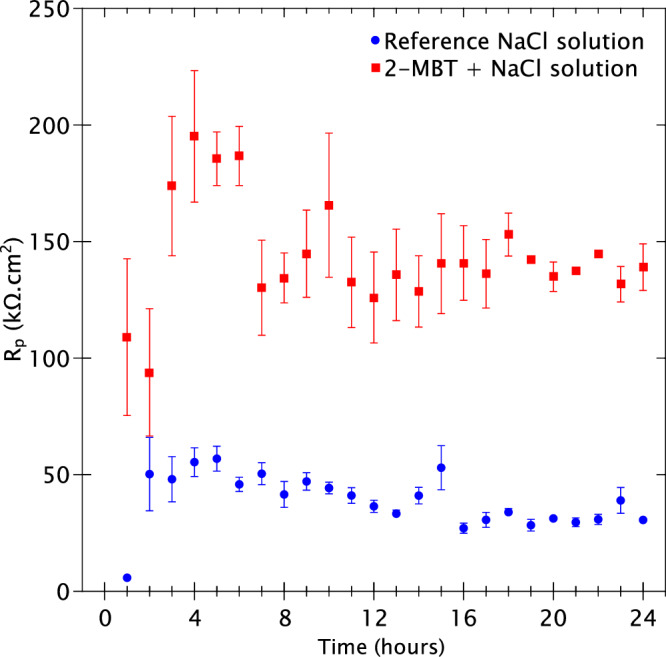
LPR measurements for the AA2024 T3 samples immersed in the reference NaCl solution and the 2-MBT-containing NaCl solution at 1-h intervals over 24 h of total experimental time.

The LPR measurements were taken at 1-h intervals to determine the polarisation resistance in each electrolyte. The benefit of using LPR is that a very narrow range of potential is applied to obtain the current flowing between working and counter electrodes thereby avoiding any variation of the interfacial properties of the sample during the measurement^38^. The results are presented in Fig. 2 with the polarisation resistance in kΩ.cm² as a function of time over 24 h experimental time.

The LPR measurements show that the polarisation resistance of the sample immersed in the reference solution is lower compared to that in the 2-MBT-containing solution. After the first hour of immersion in the reference NaCl solution, the alloy sample exhibits a very low polarisation resistance with a value of 5.8 ± 0.1 kΩ.cm². However, this increases by a factor of approximately 9 times, to 50.3 ± 15.7 kΩ.cm² after 2 h of immersion. It is important to note that the first 2 h of immersion do not always yield reliable results for LPR measurements since the OCP fluctuates significantly during early exposure and the sample has not reached a steady state yet.

The polarisation resistance remains relatively stable for the next 5 h of immersion. After 6 h of immersion, a consistent decrease in resistance is observed until 13 h indicating a gradual attack during this period, most likely the general corrosion of the alloy matrix. Between 14–15 h of immersion, a slight increase in polarisation resistance is observed, which was also found to be reproducible when the experiments were repeated. Following this, the resistance drops to 27 ± 2.2 kΩ.cm² at 16 h of immersion and remains within a range of ±3 kΩ.cm² till the end of the experiment. In previous works on aluminium alloys^39^, it has been suggested that a small spike in resistance towards the latter half of the experiment could arise due to the formation of corrosion products that deposit at the mouth of pits and other sites of anodic activity thus limiting attack for a short period. However, attack resumes once these porous corrosion products are redistributed over time, resulting in a drop of the LPR values once again.

In contrast, the sample immersed in a 2-MBT-containing NaCl solution exhibits drastically different and larger polarisation resistances over 24 h. Initially, the resistance value after 1 h of immersion is measured to be 109.0 ± 33.6 kΩ.cm², which is approximately 19 times higher than in the reference solution, indicating that 2-MBT has a positive effect against corrosion of the sample right from the start of immersion. After 2 h, there is a large increase in the polarisation resistance to 173.8 ± 29.9 kΩ.cm², which is stable until 6 h. This increase suggests that during this time the inhibitor molecules are at their peak performance in limiting the rate of corrosion reactions.

After 7 h of immersion, a drop in resistance of approximately 50 kΩ.cm² is observed, following which the polarisation resistance remains within the range of 140 ± 20 kΩ.cm² until the end of the experiment. This drop implies an increase in the rate of attack on the sample. Nevertheless, the fact that the LPR values of this inhibited sample do not go down to those of the un-inhibited sample suggests that 2-MBT is still effective as an inhibitor. Once the polarisation resistance values stabilise for both samples, approximately after 7 h, it is observed that there is a factor of 3–4 in polarisation resistance between the sample with 2-MBT versus the sample without 2-MBT for the remainder of the experiment. Therefore, the LPR measurements confirm that there is indeed an effect of corrosion inhibition of the AA2024 T3 alloy in presence of 2-MBT in the corrosive environment.

**Fig. 3:**
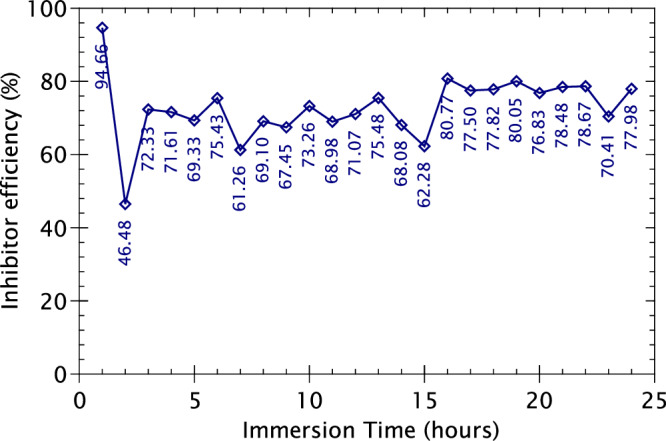
Inhibitor efficiency calculated for 2-MBT on the AA2024 T3 alloy as a function of immersion time.

A key feature of the LPR measurements is the ability to quantify inhibitor efficiency in terms of percentage from the results obtained. This metric can be obtained by using the formula given below:

1 $$\eta =\left(\frac{{R}_{p}^{{inh}}-\,{R}_{p}^{{blank}}}{{R}_{p}^{{inh}}}\right)\times 100\, \%$$

where *η* is the inhibition efficiency, *R*_*p*_^*inh*^ the mean polarisation resistance of the inhibited sample, and *R*_*p*_^*blank*^ the mean polarisation resistance of the un-inhibited sample. Using this equation, we obtain the inhibitor efficiency of 2-MBT on AA2024 T3 alloy for each hour of immersion, which is presented in Fig. 3.

The first 2 h of immersion presents highly fluctuating inhibitor efficiencies, an effect of the unstable OCP during initial hours of immersion, as discussed earlier. Once the sample reaches a steady state, it is observed that the inhibitor efficiency for 2-MBT is between 61.26 to 75.48% until 15 h of immersion, indicating a consistent performance of the inhibitor. At 16 h of immersion, a considerable jump of the inhibitor efficiency is observed from 62.28 to 80.77%, after which it remains between 70.41 to 80.05% till the end of the experimental time. This increase in inhibitor efficiency towards the end of the experiment illustrates that the performance of 2-MBT on AA2024 does not diminish over time but rather amplified over increasing immersion time. The overall inhibitor efficiency for 2-MBT on the AA2024 T3 alloy was determined to be 72.93%.

**Fig. 4:**
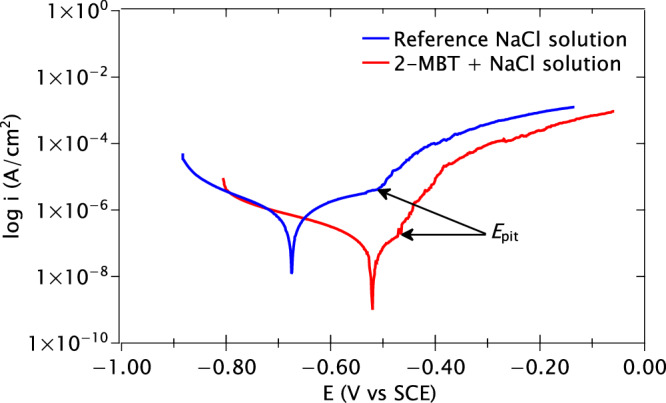
Typical potentiodynamic polarisation curves of the AA2024 T3 samples obtained after 24 h of immersion in the reference NaCl solution and the 2-MBT-containing NaCl solution.

The typical polarisation curves obtained by potentiodynamic polarisation are presented in Fig. 4. The mean corrosion potentials and the pitting potentials are given in Table 1 along with their standard error values. While the line-shape of the two polarisation curves are rather similar, the shifts in potential and current density observed for the sample in the 2-MBT-containing solution are consistent with corrosion protection. The polarisation curve in the reference NaCl solution exhibits its corrosion potential at –0.66 ± 0.02 V vs SCE, whereas the curve obtained in the 2-MBT-containing solution exhibits a nobler potential at –0.51 ± 0.02 V vs SCE, a difference of 0.15 V between them. This indicates that the AA2024 sample is less prone to corrosion attack in the presence of inhibitor, confirming the OCP and LPR measurements. This result is also in agreement with previous works on the use of 2-MBT inhibitor for the AA2024 alloy^31,33^.

**Table 1:** Corrosion potentials and pitting potentials determined for the AA2024 T3 alloy polarised in a reference NaCl solution and in a 2-MBT-containing NaCl solution from the potentiodynamic polarisation curves

	*E*_corr_ (V vs SCE)	*E*_pit_ (V vs SCE)
Reference NaCl	–0.66 ± 0.02	–0.52 ± 0.01
2-MBT-containing NaCl	–0.51 ± 0.02	–0.48 ± 0.01

The values presented here are the average of three measurements along with their standard errors.

The pitting potential of the sample polarised in the reference solution was determined to be –0.52 ± 0.01 V vs SCE, while that of the sample polarised in the 2-MBT-containing solution is –0.48 ± 0.01 V vs SCE. Therefore, the presence of 2-MBT shifts the pitting potential to a nobler value, albeit by 40 mV only, indicating a slightly increased protection by 2-MBT against localised corrosion attack. Finally, it is also evident from Fig. 4 that the corrosion current density in the reference solution is higher than that of the 2-MBT-containing solution, revealing that 2-MBT also reduces the rate of corrosion attack on the AA2024 T3 aluminium alloy.

To ensure that no changes occurred on the anodic side due to prior cathodic polarisation, anodic scans were performed from –0.05 V to +0.5 V vs OCP and the obtained anodic polarisation curves were compared with those of the entire potentiodynamic polarisation (Fig. 4). These curves, presented in Fig. S1 of the supplementary information, show no significant difference compared to the curves in Fig. 4, thus confirming that there was no effect of prior cathodic polarisation on the anodic scan of the potentiodynamic polarisation measurements.

**Fig. 5:**
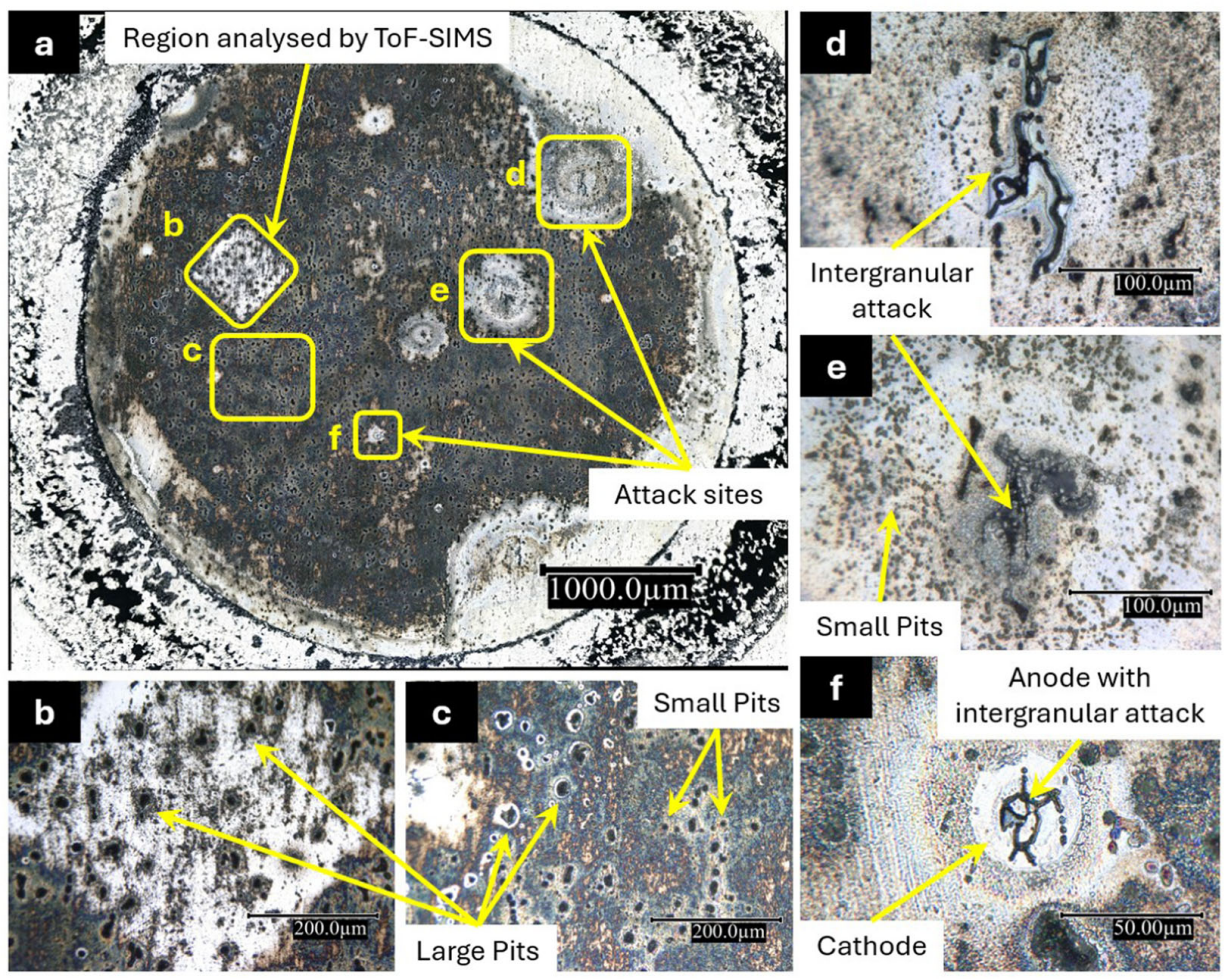
Optical microscopy images of the AA2024 T3 alloy sample after 24 h immersion in the reference NaCl solution. (**a**) Corrosion attack on the entire exposed area, (**b**) Region analysed by ToF-SIMS, (**c**) Pits formed on the surface, (**d**) and (**e**) Intergranular attack surrounded by small pits, and (**f**) Galvanic attack site with intergranular attack in the anode surrounded by a protected cathode.

**Fig. 6:**
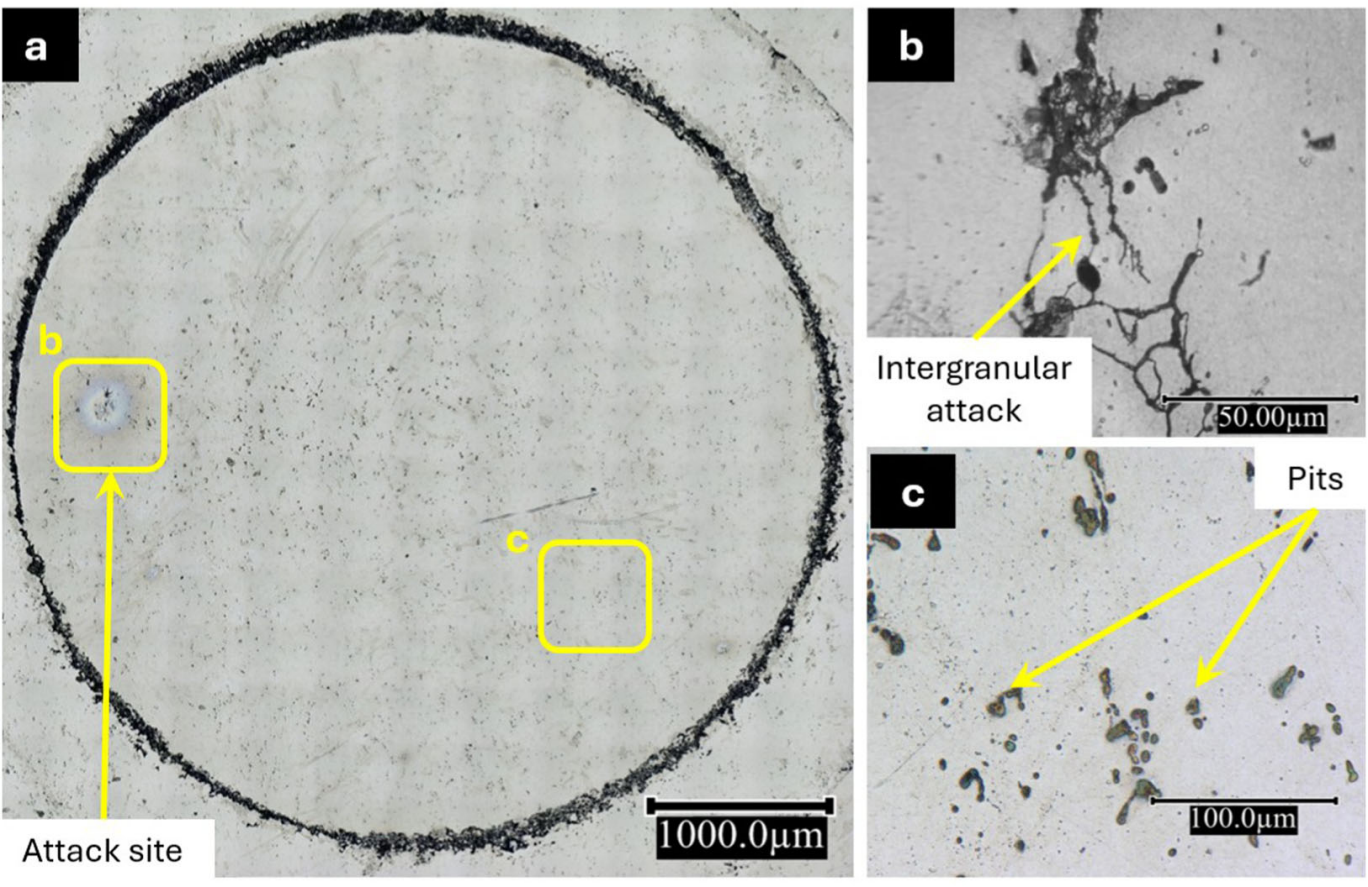
Optical microscopy images of the AA2024 T3 alloy sample after 24 h immersion in the 2-MBT-containing NaCl solution. (**a**) Corrosion attack on the entire exposed area, (**b**) Intergranular attack within the attack site, and (**c**) Pits formed on the surface.

Optical microscopy was performed ex situ after 24 h immersion at OCP conditions in either electrolyte to visually observe the corrosion morphology and the effects of 2-MBT. The OM images of the AA2024 sample immersed for 24 h in the reference and inhibitor-containing NaCl solutions are shown in Figs. 5 and 6, respectively.

Figure5a shows the entire surface of the AA2024 T3 alloy which was exposed to the reference NaCl solution during 24 h of immersion at OCP conditions. It is observed that the alloy surface is immensely attacked, showing various types of corrosion attack such as pitting corrosion, intergranular corrosion, and local attack sites. The attack sites, ranging in size from 125 to 730 µm in diameter, are mostly within an enclosure of reduced corrosion attack. Some of these attack sites have been enlarged in Fig.5d-f. These are indicative of local galvanic coupling where the main attack region is the anode and the region around it the cathode. This morphology of corrosion attack on aluminium alloys has also been reported by others^40–42^.

Figure5b shows the region which was analysed by ToF-SIMS chemical mapping and the images are presented later in Fig.13. The pits formed on the surface of the alloy sample are seen clearly in Fig.5c. These pits range in size from sub-micrometre pits up to 24.6 µm in diameter. The attack sites enlarged in Figs.5d-4f all exhibit intergranular attack at their centres. Encircling these regions of intergranular attack is an area exhibiting attack to a lesser degree, characterised by smaller pits ( <3 µm in diameter), than that of the remaining alloy surface. This is because they act as cathodes with respect to the intergranular attacked region, which acts as an anode, thereby largely protecting them from severe corrosion attack. This feature is clearly seen in Fig. 5f where the anode and cathode of the local galvanic couple form a complete attack site. Nevertheless, it is evident that after 24 h immersion in the 0.01 M NaCl solution, the alloy sample is severely corroded on the entire surface. While the surface is covered in pits, as the dominant attack morphology, along with local attack sites exhibiting intergranular corrosion, the scale of the corrosion attack qualifies it to be generalised corrosion. This is also why we observe a (critical) pitting potential for this sample from the potentiodynamic polarisation curves (Fig.4) at a higher value compared to the corrosion potential of the sample.

Figure6a shows the entire surface area exposed to the 2-MBT-containing NaCl solution during 24 h of immersion. The inhibiting effect of 2-MBT observed from these images is significant. While pits are observed on the surface, they are somewhat smaller than those seen in Fig. 5. Moreover, only one attack site (enlarged in Fig. 6b) is seen on this entire exposed surface. Like for the sample immersed in the reference NaCl solution, the morphology of attack within this region is a network of intergranular attack. However, a cathodic region surrounding this intergranular attack (anodic region) is not evident, as it was for the sample immersed in the reference solution. While this could be due to the smaller attack site and lower corrosion rate on this sample (Table 1), another explanation could be that the entire surface surrounding it acts as the cathode due to the presence of 2-MBT molecules. Figure 6c shows the pits on the surface of this sample, enlarged for better characterisation. They are smaller, maximum 15 µm in diameter, compared to those found on the sample immersed in the reference NaCl solution. Comparing the two samples, it can be concluded that 2-MBT inhibits corrosion significantly on the AA2024 T3 alloy in neutral chloride conditions.

### Surface State after Mechanical Polishing

A prerequisite to understand the changes of the AA2024 T3 alloy surface, induced by immersion in the NaCl solution and the 2-MBT inhibition effects, is to characterise the initial surface state prior to exposure to the solutions. Therefore, a mechanically polished AA2024 T3 sample was analysed by XPS and ToF-SIMS.

**Fig. 7:**
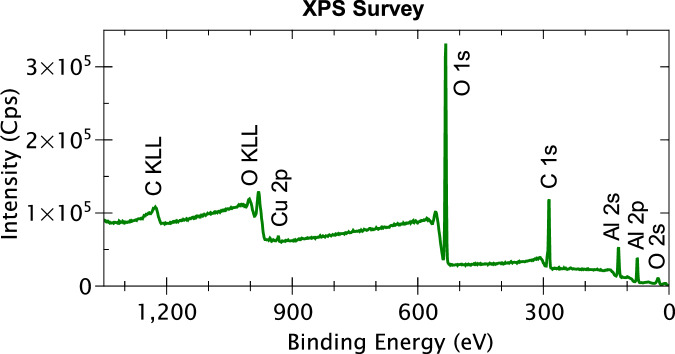
XPS survey spectrum for the mechanically polished AA2024 T3 alloy sample.

**Fig. 8:**
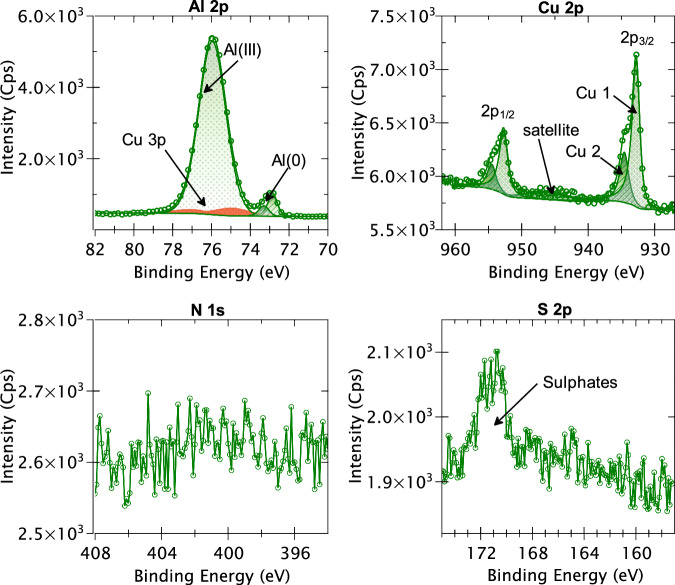
XPS Al 2p, Cu 2p, N 1s, and S2p core level spectra obtained for a mechanically polished AA2024 T3 alloy sample.

The XPS survey spectrum obtained for the mechanically polished AA2024 T3 alloy is presented in Fig. 7. The discernible peaks associated with specific elements have been identified based on their BE values. It is observed that apart from aluminium, copper, oxygen, and carbon, no other element can be identified from the survey spectrum. However, since some of the alloying elements may have low intensities which are not detected in the survey spectrum, high-resolution core level spectra of all the alloying elements (shown in Table 2) of the AA2024 T3 alloy were taken along with the N 1s and S 2p spectra. Figure 8 presents the XPS high-resolution spectra of Al 2p, Cu 2p, N 1s, and S 2p core levels obtained for the mechanically polished AA2024 T3 alloy sample. Since the signal-to-noise ratio was too low for the Fe 2p, Mg 2p, Mn 2p, Si 2p, Ti 2p, Zn 2p, Zr 3d core levels, and the Cu LMM Auger spectrum, they are not presented here.

**Table 2:** Chemical composition of the AA2024 T3 alloy given in weight percentage

Element	Cu	Mg	Mn	Zn	Fe	Si	Zr	Ti	Al
wt. %	3.70 ‒ 4.50	1.20 ‒ 1.50	0.15 ‒ 0.80	0.25	0.20	0.15	0.10	0.15	Bal.

The Al 2p spectrum exhibits two distinct components at 72.9 eV BE and at 75.9 eV BE. The component at 72.9 eV is assigned to metallic aluminium (Al(0)) and was decomposed using its 2p_3/2_ – 2p_1/2_ spin-orbit doublet with a branching ratio of 0.5 and splitting of 0.44 eV^43^. An Asymmetric Lorentzian (LA) line-shape convoluted with a Gaussian was used for the fitting of this metallic peak with a full width at half maximum (FWHM) of 0.5 eV. The component with the higher intensity at 75.9 eV BE was identified as the Al(III) species^43^, usually assigned to aluminium oxide and/or hydroxide, and decomposed using a Gaussian/Lorentzian product line shape. This component was not decomposed into its spin-orbit doublets due to significant overlapping of the doublets and its large FWHM (1.6 eV). It is important to note that within this Al(III) peak, there is an overlap of the Cu 3p core level which can result in an overestimation of the Al(III) intensity. To correct this overestimation, the Cu 3p component was fitted by taking the intensity of the Cu 2p component and normalising it with the photoionization cross section, the transmission function of the analyser, and the inelastic mean free path. Once the intensity of the Cu 3p component was determined, it was decomposed into its 3p_3/2_ – 3p_1/2_ spin orbit doublet at 74.9 and 77.0 eV BEs, respectively, with a branching of 0.5 using an LA line-shape^43^. The contribution of this Cu 3p component was found to be 7.9% of the intensity of the Al(III) peak.

The Cu 2p spectrum exhibits its 2p_3/2_ and 2p_1/2_ spin-orbit doublets with a split of 19.9 eV, as expected for the Cu 2p core level^43^. The 2p_3/2_ component was decomposed into two peaks – Cu 1 at 932.8 eV and Cu 2 at 934.7 eV. The Cu 1 peak corresponds to both metallic copper and copper (I) oxide, i.e., Cu_2_O^24,44,45^. The second component, Cu 2, is most likely reminiscent of the Cu(OH)_2_ species^46–48^, which is also indicated by the presence of a small satellite peak at 944 eV. It is not possible to further decompose the peaks to distinguish between metallic copper and Cu_2_O, since the difference in BE between these two chemical states is only 0.1 eV^43^. In previous works^24–26,37^, the Cu LMM Auger spectra has been used for this purpose, thereby giving us a ratio between the Cu(0), Cu(I), and Cu(II) components. However, due to the low intensity of Cu overall, the signal-to-noise ratio of the Cu LMM Auger spectrum obtained for this sample was too low for confident fitting.

The N 1s range of BE does not show any discernible peak here indicating that nitrogen is below the detection limit of our XPS instrument (<1 at.%). On the contrary, the S 2p spectrum exhibits a peak at 171.1 eV BE, albeit with very low intensity. This peak most likely corresponds to sulfate species^43^, which are quite common contamination species found on aluminium. Nevertheless, since the BE of this species is higher than what is expected for the sulfur from the 2-MBT molecule (161.9–164.4 eV)^24,49,50^, it should not cause too many issues for analysis after 2-MBT molecule adsorption.

**Fig. 9:**
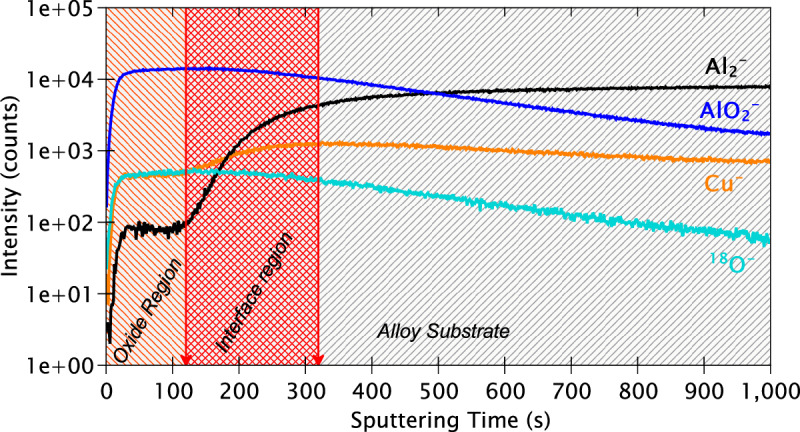
ToF-SIMS depth profiles for the mechanically polished AA2024 T3 alloy sample.

The ToF-SIMS in-depth profiles obtained for a mechanically polished sample are shown in Fig. 9. Each plotted ion profile is characteristic of certain species in the alloy, as determined by the mass of these species. The Al_2_^‒^ ion (53.9674 amu) was selected as mostly characteristic of aluminium metal and the AlO_2_^‒^ ion (58.9763 amu) as mainly representative of aluminium oxide. The Cu^‒^ ion (62.9264 amu) was selected as representative of metallic copper. This is in line with previous works which have also used these ions for identification of the metallic and oxidised Al and Cu species^51,52^. However, it must be kept in mind that the oxidised ions of an element can be representative of both the metallic and oxidised species due to the high affinity of elements towards oxygen in the residual gas of the instrument. Finally, the ^18^O^‒^ ion profile (17.9997 amu) was selected to represent the overall oxides on the substrate since the O^‒^ ion (15.995 amu) saturates the detector due to its high intensity. The ion species relating to Fe, Mg, Mn, Si, and their respective oxides are not shown due to their low intensities. Instead, they have been shown in the chemical mapping images and are interpreted thereafter.

The line-shapes of the ion depth profiles in Fig. 9 indicate that there are three distinct regions, as marked. The first and outermost region is the region where the AlO_2_^‒^ and ^18^O^‒^ ions exhibit their maximum intensities while the Al_2_^‒^ and Cu^‒^ ions exhibit much lower intensities. Therefore, this region is assigned to the oxide layer covering the alloy sample. The maxima of the AlO_2_^‒^ ion coincides with the start of increase of Al_2_^‒^ ion intensity, as indicated by the first red line at 120 s of sputtering time, thereby signifying the end of the oxide layer region.

Following 120 s of sputtering time, the intensities of both the AlO_2_^‒^ and ^18^O^‒^ ion profiles start to decrease, while those of the Al_2_^‒^ and Cu^‒^ ions progressively increase. This concurrent decrease of the oxide signals and the increase of the metallic signals indicate that this region is the interfacial region between the oxide layer and the metallic substrate. The Cu^‒^ ion reaches its maximum intensity at 320 s, which marks the end of the interfacial region and the start of the metallic substrate region, as indicated by the second red line.

After 320 s of sputtering time, the Al_2_^‒^ ion profile intensity continues to increase even after the Cu^‒^ ion intensity starts to decrease. This indicates that, within the metallic substrate, copper is located above aluminium in terms of depth from the surface. This appears consistent with selective oxidation of aluminium of the polished matrix exposed to air, leaving metallic copper enriched at the interface between the oxide layer and the substrate^53^. Be that as it may, this effect could also be due to differential sputtering of the alloying elements during the sputtering process in ToF-SIMS, which must not be discounted, and would result in copper being sputtered at a slower rate than aluminium.

**Fig. 10:**
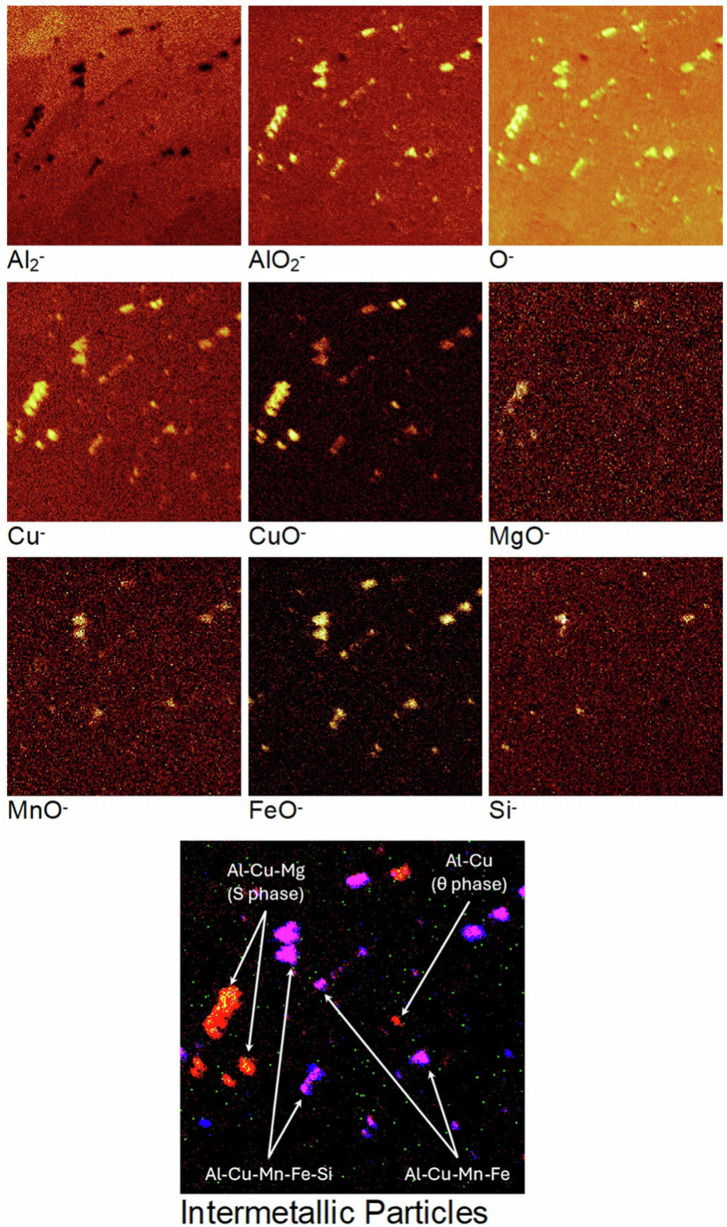
ToF-SIMS chemical maps obtained for the mechanically polished AA2024 T3 alloy sample over 1000 s of sputtering time. The total area analysed is 200 × 200 μm². The brighter zones correspond to regions of higher intensities.

To observe the minor alloying elements within the alloy and to characterise the intermetallic particles, the ToF-SIMS chemical maps were recorded for the mechanically polished AA2024 T3 sample. These 2-D images, presented in Fig. 10 with their individual intensity scales, were obtained by summing stacks of 2-D images of the ion species recorded at each measurement interval over the entire analysed depth corresponding to 1000 s of sputtering time. Therefore, the images shown in Fig. 10 correspond to the sum of the ion species intensity over 1000 s of sputtering.

The chemical maps of the AlO_2_^‒^ and the O^‒^ ions exhibit high intensities overall, confirming the presence of an oxide film on the surface. The intermetallic particles present in the AA2024 T3 alloy sample are also clearly observed from the chemical maps. While these particles are of varying sizes and shapes, they can broadly be classified into larger and smaller particles. Both categories of particles exhibit concurrence in the aluminium and copper chemical maps (metal and oxide ions). It is important to note that the Al_2_^‒^ ion image shows black voids at the IMP sites, which is due to the higher concentration of Al in the matrix than in the particles.

A few of the larger particles correlate with the MgO^‒^ ion image indicating that these particles are the Al-Cu-Mg (S phase) particles. The MnO^‒^ and FeO^‒^ ion images show rather reliable unanimity with the remaining large particles observed in the Cu^‒^, CuO^‒^, AlO_2_^‒^, and O^‒^ images. These particles were identified as the Al-Cu-Fe-Mn particles. In some of these particles, the presence of silicon is observed as well from the Si^‒^ image, indicating that some of them are in fact the Al-Cu-Fe-Mn-Si particles. The smaller particles observed in the Cu^‒^, AlO_2_^‒^, and O^‒^ images do not exhibit any concurrence with the other ion images, indicating that they are most likely Al-Cu (θ phase) particles. The different intermetallic particles identified on this sample are shown clearly on the overlay image, where the Al-Cu-Mg (S phase) particles are orange, the Al-Cu (θ phase) particles are red, and finally the Al-Cu-Mn-Fe-(Si) particles are pink in colour.

The overall intensities of the MgO^‒^, MnO^‒^, FeO^‒^, and Si^‒^ ion images are very low, especially in the matrix due to their presence as minor alloying elements in the alloy with very low concentrations.

### Surface Analysis after Corrosion In Reference NaCl solution

**Fig. 11:**
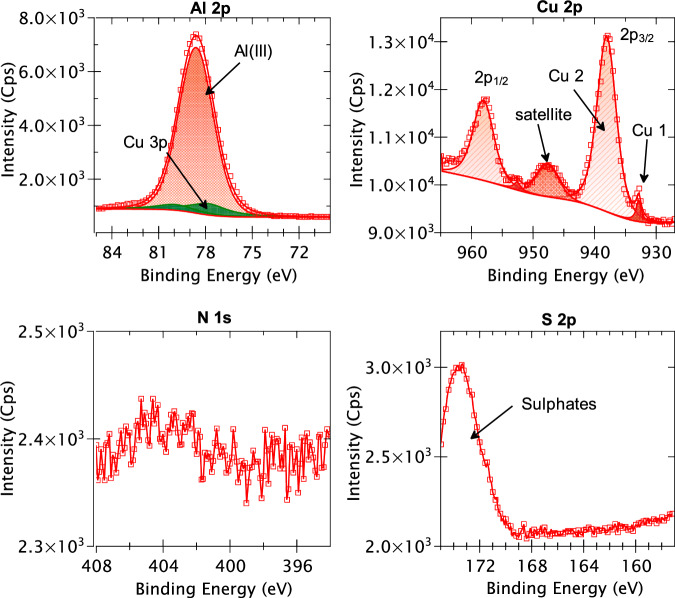
XPS Al 2p, Cu 2p, N 1s, and S2p core level spectra obtained for the AA2024 T3 alloy sample after 24 h immersion in the reference NaCl solution.

To understand the mechanisms of inhibitor action, one also needs to understand what would occur in the absence of the inhibitor, i.e., the effect of corrosion attack on AA2024 in a neutral chloride solution. This effect is investigated by surface analysis using XPS and ToF-SIMS. Figure 11 presents the XPS spectra of the Al 2p, Cu 2p, N 1s, and S 2p core levels of the A2024 T3 alloy sample after 24 h immersion in the reference NaCl solution.

The Al 2p spectrum exhibits only one large peak which corresponds to the Al(III) chemical state, i.e., aluminium oxides/hydroxides. Similar to the XPS spectra in Fig. 8, the contribution of the Cu 3p_3/2_ – 3p_1/2_ spin orbit doublet within this Al 2p spectrum was fitted based on the Cu 2p intensity. Comparing with the Al 2p spectrum of the polished alloy sample, there are two main differences: the first difference is the absence of the metallic Al peak, while the second difference is the positive BE shift for the Al(III) component. Both these differences can be traced back to the corrosion of the alloy resulting in the formation of a relatively thick surface layer of corrosion products, which is aluminium hydroxide in this case^54^. The thickness of this layer is larger than the information depth of the XPS analysis, i.e., greater than five times the inelastic mean free path (5λ), thus resulting in the intensity of metallic aluminium being fully attenuated. This means that the metallic aluminium is buried under the thick corrosion product layer. As the oxide/hydroxide layer grows thicker, there is increased charging on the sample due to poor conductivity of this layer with the XPS analyser, thereby resulting in a positive BE shift (+ 2.7 eV) in the present case.

The Cu 2p spectrum in Fig. 11 is also substantially different from that observed for the polished sample (Fig. 8). While the Cu 1 component corresponding to metallic copper and copper (I) oxide is still observed at 932.9 eV BE, the Cu 2 component here is of much higher intensity and occurs at a 938.0 eV BE. Moreover, the presence of a large satellite peak is also observed at 947.7 eV. While the exact nature of this Cu 2 peak is not known, it most likely corresponds to the remaining copper fragments and their oxidised species within the pits and attack sites after dealloying of the IMPs (dissolution of more active elements). This would also explain the charging effect resulting in a positive shift of the BE, similar to that observed for the Al(III) component in the Al 2p spectrum.

The N 1s and S 2p spectra for the NaCl immersed sample were also recorded to investigate if there are any effects that need to be considered when analysing the 2-MBT-NaCl immersed sample (Fig. 11). The N 1s BE range does not exhibit any distinct peak, and the intensity is negligible due to the low signal-to-noise ratio. On the other hand, a large peak is observed in the S 2p spectrum at 173.6 eV BE. The positive BE shift observed for this peak is consistent with that for the Al(III) species in the Al 2p spectrum and the Cu 2 component in the Cu 2p spectra. Therefore, this peak is assigned to the presence of sulfate contaminants on the sample surface. The positive shift in BE observed here for the sulfate contaminants is caused by the presence of a thicker Al oxide/hydroxide layer on this sample. This thicker layer insulates the sulfate species, leading to a charging effect that shifts the detected binding energy to higher values.

**Fig. 12:**
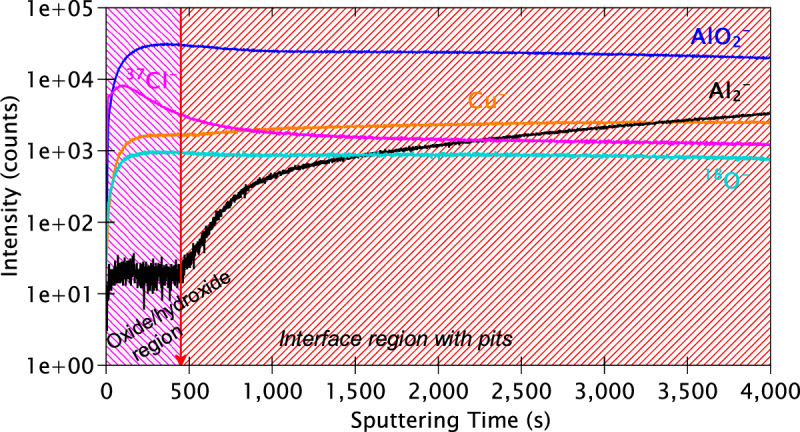
ToF-SIMS depth profiles of the AA2024 T3 alloy sample after 24 h immersion in the reference NaCl solution.

Figure 12 presents the ToF-SIMS in-depth profiles obtained on the alloy sample after 24 h immersion in the reference NaCl solution. Unlike for the polished surface (Fig. 9), there are only two distinct regions here. The first region, from 0 to approximately 450 s of sputtering time, corresponds entirely to the oxide/hydroxide region. Within this region, the intensity signal of the Al_2_^‒^ ion profile is rather low while those of the AlO_2_^‒^ and ^18^O^‒^ ion profiles are high. While a rather high intensity for the ^37^Cl^‒^ ion profile, characteristic of chloride ions (36.9660 amu), is observed throughout the 4000 s of sputtering time, an explicit peak is observed within this outer layer indicating that chloride penetration is the highest in the oxide/hydroxide region but remains substantial deeper into the sample.

After 450 s of sputtering time, a progressive increase for the Al_2_^‒^ and Cu^‒^ ion profiles is observed concurrently with a decrease for the AlO_2_^‒^ and ^18^O^‒^ ion profiles. This marks the start of the interfacial region between the oxide/hydroxide region and the modified alloy substrate, which includes pits that were formed during the 24 h of immersion. In contrast with the polished sample, the interfacial region here extends for a rather long duration of sputtering. Another noteworthy point is that instead of sharp increases/decreases, relatively slow and moderate increases/decreases are observed. This phenomenon is related to the roughness of the analysed sample due to the formation of pits, as seen in Fig. 5, thereby resulting in smoother profiles here.

**Fig. 13:**
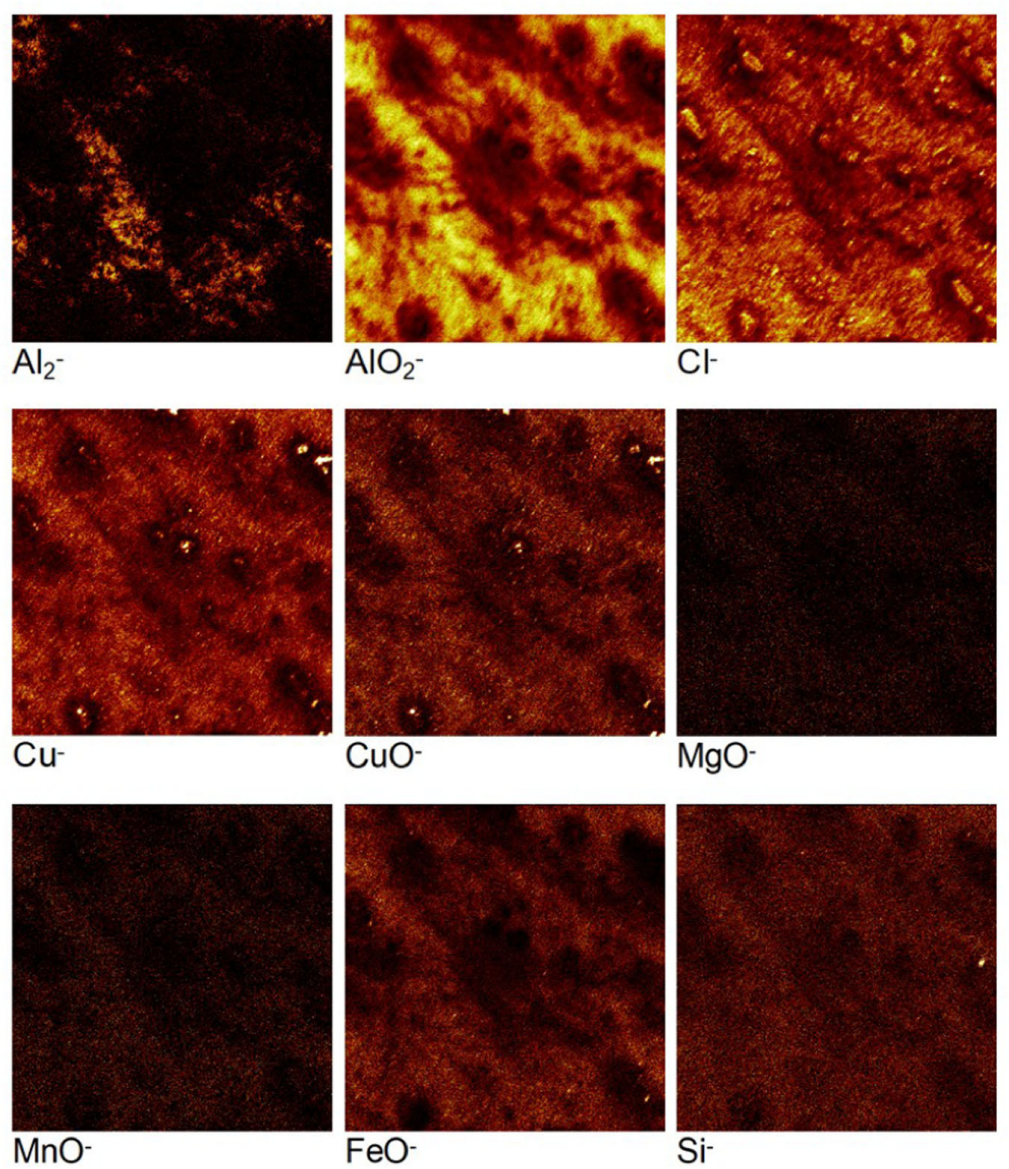
ToF-SIMS chemical maps obtained for the AA2024 T3 alloy sample after 24 h immersion in the reference NaCl solution over 1500 s of sputtering time. The total area analysed is 200 × 200 μm². The brighter zones correspond to regions of higher intensities.

**Fig. 14:**
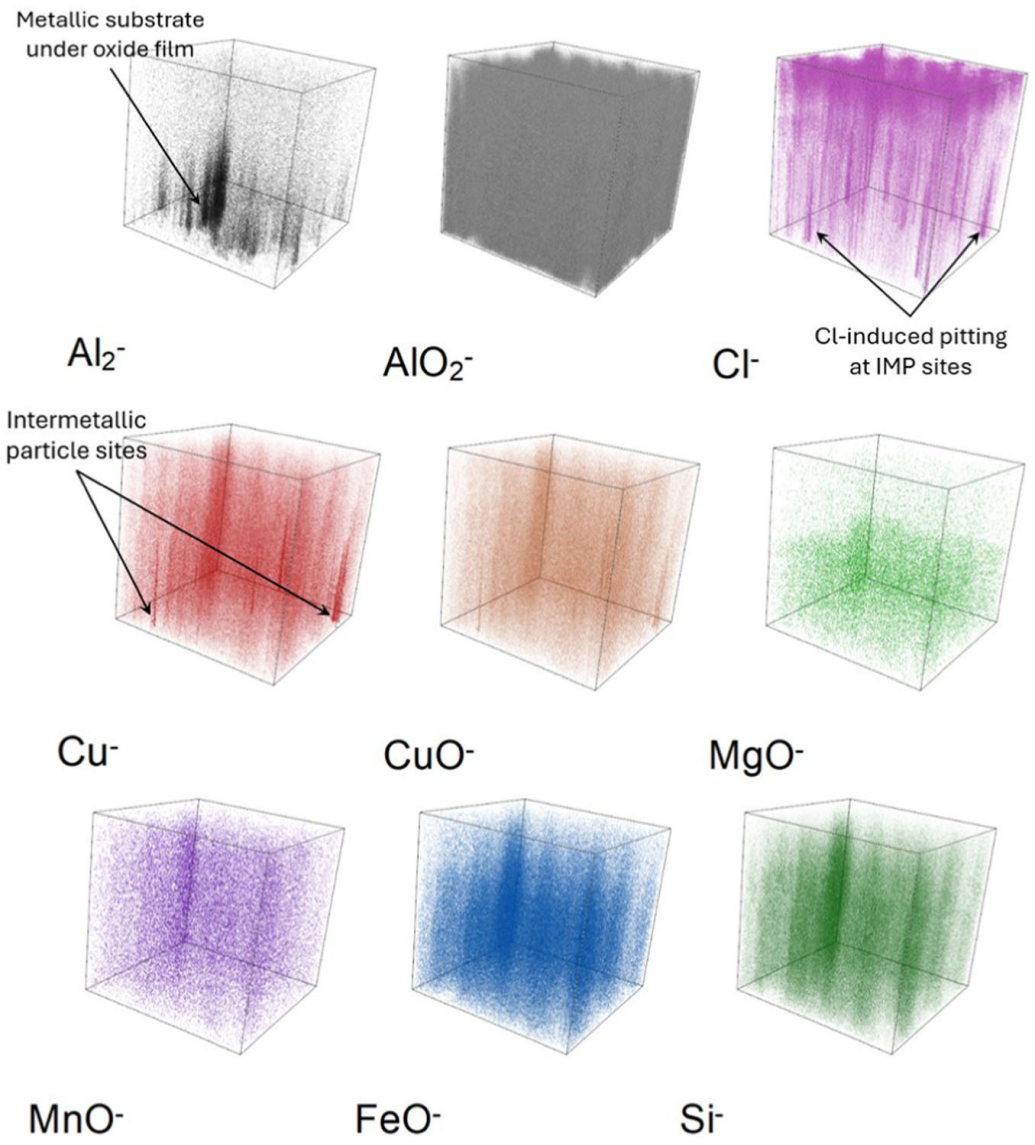
3-D reconstruction of the chemical maps obtained for the AA2024 T3 alloy sample after 24 h immersion in the reference NaCl solution over 1500 s of sputtering time. The top face corresponds to 0 s of sputtering time and the total area analysed is 200 × 200 μm².

The AlO_2_^‒^ and ^18^O^‒^ ion profiles exhibit a plateau from approximately 1000–2500 s of sputtering followed by a slow decrease till the end of the experiment. This plateau is most likely due to the presence of corrosion products deep within the pits that are found in the interfacial region. The Al_2_^‒^ ion profile, on the other hand, exhibits a continuous and gradual increase in intensity till the end of the experiment, suggesting that even after 4000 s the uncorroded substrate is not reached. Due to the extremely low concentration of the alloying elements and the surface state of the corroded sample, it is not possible to extract further information from this depth profile. Instead, we examine the chemical maps and the 3-D reconstruction also obtained by ToF-SIMS which are shown in Figs. 13 and 14, respectively.

The Al_2_^‒^ image and the 3-D reconstruction show very little intensity of metallic aluminium, mostly originating from underneath the oxide/hydroxide surface layer regions that are not attacked locally. On the other hand, the AlO_2_^‒^ image exhibits a very high intensity throughout the analysed depth (1500 s), observed by the increased brightness of its chemical map and the consistency of the colour gradient in its 3-D image. The Cl^‒^ image pinpoints the locations of the numerous pits formed during exposure to the chloride solution with higher intensities at the pit sites. Meanwhile, its 3-D image shows that the penetration of chloride ions is observed for the entire analysed depth, as mentioned earlier.

The Cu^‒^ and CuO^‒^ images exhibit several localised copper-rich regions. However, very little correlation is observed for these Cu-rich regions with the MgO^‒^, MnO^‒^, FeO^‒^, or Si^‒^ images. Moreover, these Cu-rich regions exhibit good correlation with the sites of high Cl^‒^ ion intensity. This indicates that the IMPs have undergone corrosion attack, i.e., dealloying of the particles due to the influence of chloride ions. This results in the dissolution of the active elements within the particles, thereby leaving only remnants of the more noble copper at these sites. This is further evidenced by the homogeneous distribution of these ions (MgO^‒^, MnO^‒^, FeO^‒^, and Si^‒^) over the analysed volume compared to the polished sample, indicating that these species are uniformly distributed within the analysed volume instead of locally concentrated at a particular region.

### Surface Analysis after Corrosion Inhibition by 2-MBT

**Fig. 15:**
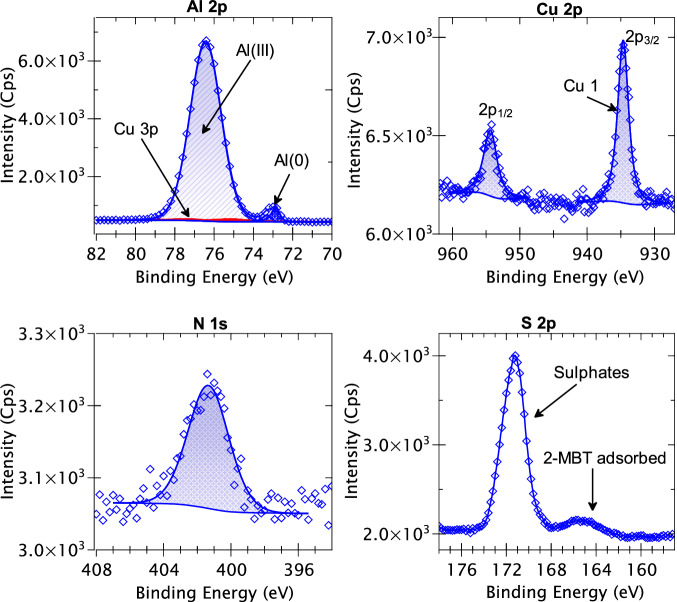
XPS Al 2p, Cu 2p, N 1s, and S2p core level spectra obtained for the AA2024 T3 alloy sample after 24 h immersion in the 2-MBT-containing NaCl solution.

The XPS Al 2p, Cu 2p, N 1s, and S 2p core level spectra of the AA2024 T3 sample after 24 h of immersion in a 2-MBT-containing NaCl solution are presented in Fig. 15. The Al 2p spectrum for this sample bears greater resemblance to that of the polished sample (Fig. 8) than after immersion for 24 h in the reference NaCl solution (Fig. 11). While the peak corresponding to metallic Al is smaller than the metallic Al peak of the mechanically polished sample, it is still detected here, unlike after exposure to the reference NaCl solution. Nevertheless, the peak corresponding to Al(III) component (oxide/hydroxide) appears at a slightly higher BE (+0.4 eV) compared to that in the polished sample. This charging effect suggests the presence of a thicker oxide/hydroxide layer for this sample.

The Cu 2p spectrum exhibits only one component for the 2p_3/2_ spin-orbit, located at 934.7 eV BE. Since there is no satellite peak observed here, we can exclude the possibility of any Cu(II) species and assign the component to Cu(0) and Cu(I) species. This means that there is a positive shift of 1.9 eV for this component compared to that observed for the polished sample in Fig. 8. While both the Al (III) component in the Al 2p spectrum and the Cu 1 component in the Cu 2p spectrum exhibit a positive shift in BE, the shift is much larger for the Cu 1 component (+1.5 eV). This indicates that the cause of the charging effect is different for the Cu 1 component from that of the Al (III) component. Since we did observe pits on the surface of the sample immersed in 2-MBT containing NaCl solution in Fig. 6, it is likely that the IMPs are partially dealloyed and are encased in corrosion products of the dealloyed elements. This phenomenon, also observed in other works^34^ and confirmed later by the ToF-SIMS chemical mapping experiments, would explain the larger shift in BE observed in the Cu 2p spectrum compared to that in the Al 2p spectrum.

The N 1s spectrum shows the presence of a peak at approximately 401.3 eV BE. This peak is attributed to the presence of adsorbed 2-MBT, since it was not observed on the polished sample or on the sample immersed in the reference NaCl solution. Considering that the BE of the Cu component is shifted by +1.9 eV, a similar shift is observed here for the nitrogen peak. Consequently, the corrected BE, after taking the shift into account, would be 399.4 eV, which corresponds to nitrogen from 2-MBT bonded to copper oxide or in the form of multi-layers, as seen in previous works^24–26,49,50^.

Finally, the S 2p spectrum in Fig. 15 shows a large peak at 171.1 eV corresponding to sulfates. While it is more intense than that observed on the polished sample, this peak is also of higher intensity than that observed on the sample immersed in the reference NaCl solution. A small peak is observed at lower BE with its full width at half maximum (FWHM) spanning across 5–6 eV. This peak corresponds to approximately 10 – 13% of the total sulfur intensity and is attributed to the presence of 2-MBT. Due to insufficient resolution and low signal to noise ratio, this peak has not been decomposed and deductions from this spectrum have been made keeping this point in consideration.

Considering the BE values of sulfur from 2-MBT adsorbed on pure copper from previous works^24–26,45,49,50^, the 2p_3/2_ components were measured at 161.9 eV for S bonded to metallic Cu, 162.8 eV for exocyclic S from 2-MBT, and 164.2 eV for endocyclic S from 2-MBT. The latter two components correspond to the molecule adsorbed on copper oxides and/or in the form of multi-layers. Taking into account that a positive BE shift of +1.9 eV is observed for the nitrogen peak and the copper peak, a comparable shift is observed here for the peak attributed to 2-MBT adsorbed, thus confirming our assignment of the peak. The presence of adsorbed 2-MBT also explains the increase in intensity of the sulfate peak here, as we have previously also observed the formation of sulfates on pure copper in neutral chloride conditions as a by-reaction occurring during 2-MBT adsorption^26^.

**Fig. 16:**
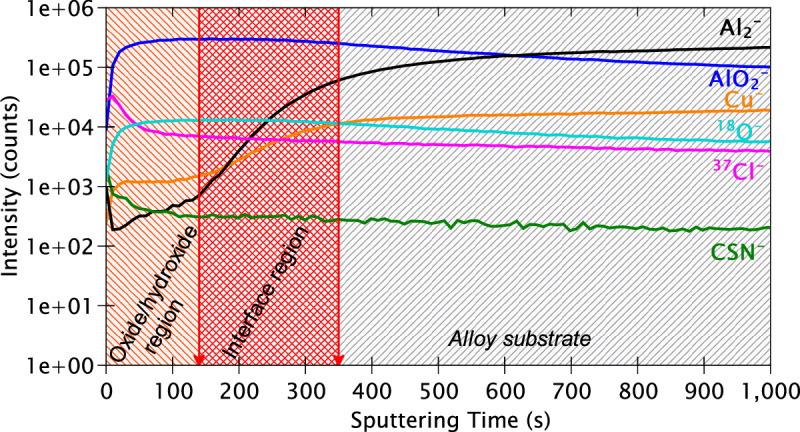
ToF-SIMS depth profiles of the AA2024 T3 alloy sample after 24 h immersion in the 2-MBT-containing NaCl solution.

The ToF-SIMS in-depth profiles obtained for the AA2024 T3 alloy sample immersed for 24 h in the 2-MBT-containing NaCl solution are presented in Fig. 16. Clearly, these depth profiles resemble those of the polished sample (Fig. 9) rather than those of the sample immersed in the reference NaCl solution (Fig. 12). Three distinct regions are observed once again – the outer oxide/hydroxide region (0–140 s), the interfacial region between oxide and substrate (140–350 s), and finally the alloy substrate region (350 s onwards). The oxide and interfacial regions for this sample are only slightly larger than those of the polished sample, confirming that 2-MBT restricts oxidation of the alloy. This is also consistent with the XPS Al 2p spectrum still showing a metallic component (Fig. 15).

The AlO_2_^‒^ and ^18^O^‒^ ion profiles exhibit a high intensity initially, reaching their maxima at approximately 140 s, followed by a slow decrease with increasing sputtering time. As their intensities decrease, increases in the intensities of Al_2_^‒^ and Cu^‒^ ions are observed, indicating the end of the outer oxide/hydroxide region and the start of the interface region. At around 350 s, the Cu^‒^ ion profile starts to level off before the Al_2_^‒^ ion profile, consistent with the trends observed for the polished sample. The ^37^Cl^‒^ ion profile shows that while its intensity is the highest on the top-most surface, it decreases sharply within the oxide/hydroxide layer and is followed by a more gradual decrease from 90 s of sputtering onwards. The occurrence of sharper profiles for this sample suggests that the sample is not severely damaged due to corrosion attack as the sample immersed in the reference NaCl solution, as seen from the OM images (Fig. 6). However, the extremely slow decrease of the ^37^Cl^‒^ ion profile from 350–1000 s indicates that chloride ions are still present within the alloy substrate region of the profile, possibly in deeper pits, as shown later in the ToF-SIMS 3-D images.

**Fig. 17:**
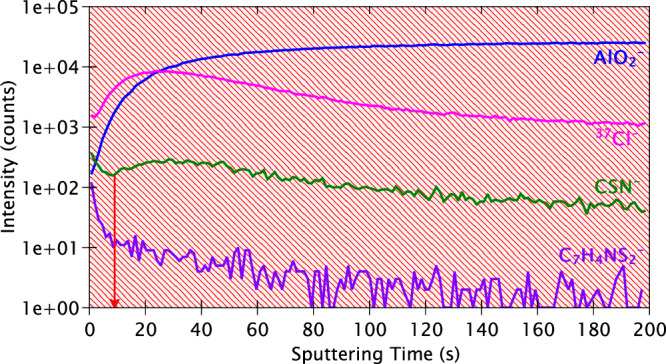
ToF-SIMS depth profiles of the top-most surface of the AA2024 T3 alloy sample after 24 h immersion in the 2-MBT-containing NaCl solution.

Finally, if we consider the profile of the CSN^‒^ ion (57.9738 amu), which is characteristic of the 2-MBT molecule fragment, a higher intensity is observed on the top-most surface which is followed by a very sharp decrease within the first 20 s of sputtering. The intensity continues to decrease, although more gradually, till approximately 90 s of sputtering time. This suggests that the inhibitor is adsorbed on the top-most surface and is easily sputtered away. To obtain a better time resolution of our measurements, the sputtering current and energy for the Cs^+^ beam were reduced and analysis was repeated in a different location of the sample surface. The depth profiles of the extreme surface of this sample (with 2-MBT) are presented in Fig. 17.

While the CSN^‒^ ion is considered as a fragment of the 2-MBT molecule, the C_7_H_4_NS_2_^‒^ ion (165.9811 amu) is selected as representative of the entire 2-MBT molecule, as in prior works^24–26^. The AlO_2_^‒^ and the ^37^Cl^‒^ ion profiles are displayed as a reference for the top-most surface. It is observed that both the ion species relating to the inhibitor molecule exhibit a sharp decrease in intensity for a very short period of sputtering, from the top-most surface (0 s) to approximately 10 s, indicating that a thin layer of 2-MBT molecules is adsorbed on the top-most surface of the sample.

**Fig. 18:**
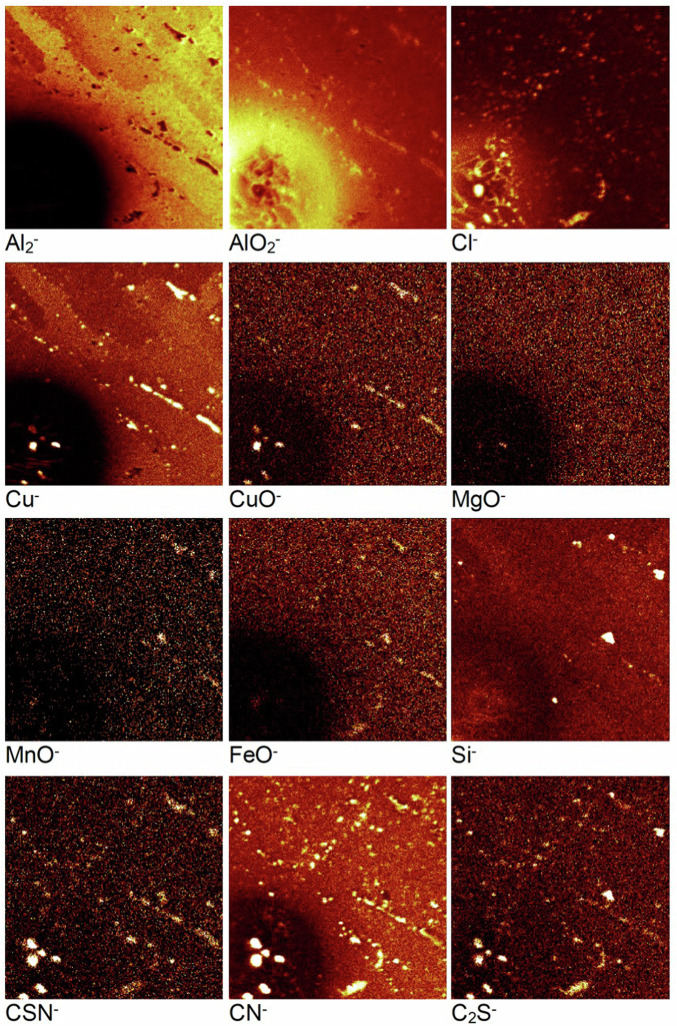
ToF-SIMS chemical maps obtained for the AA2024 T3 alloy sample after 24 h immersion in the 2-MBT-containing NaCl solution over 1000 s of sputtering time. The total area analysed is 160 × 160 μm². The brighter zones correspond to regions of higher intensities.

**Fig. 19:**
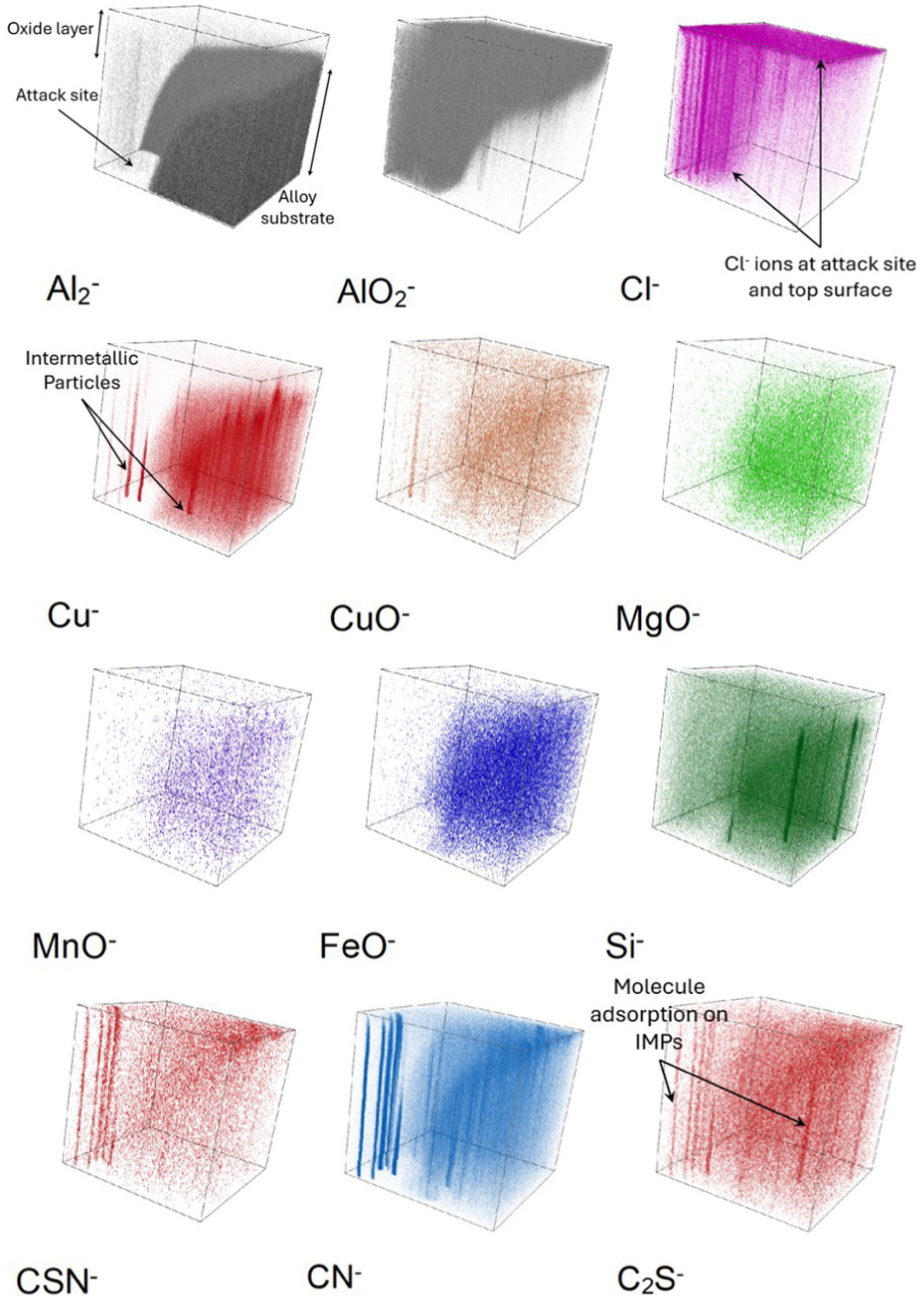
3-D reconstructed images obtained for the AA2024 T3 alloy sample after 24 h immersion in the 2-MBT-containing NaCl solution over 1000 s of sputtering time. The top face corresponds to 0 s of sputtering time and the total area analysed is 160 × 160 μm².

Following this sharp decrease, the C_7_H_4_NS_2_^‒^ ion exhibits a gradual decrease in intensity till approximately 80 s, where the intensity drops to 0 and only experimental noise is observed. On the other hand, the CSN^‒^ ion profile exhibits an inner wave from 10–60 s. This inner wave could arise from 2-MBT molecules adsorbed on the IMPs. However, to be conclusive, we must study the chemical maps of the analysed volume as done above. These chemical maps and their 3-D reconstructed images obtained over 1000 s of sputtering time are presented in Figs. 18 and 19, respectively. The area that was analysed was selected so as to include a region of attack, i.e., a pit site, along with a region where attack was not apparent.

The Al_2_^‒^ image shows a large black void at the bottom left corner. This region is an attack site formed due to corrosion attack as confirmed with the AlO_2_^‒^ map and its 3-D image. It shows that the region around this attack site is rich in aluminium oxide/hydroxide, which is consistent with the formation of corrosion products around pits and trenches. Considering the Cl^‒^ map, a very high localisation of the intensity is observed within the attack site, however, a much lower intensity is observed on the rest of the analysed area compared to the previous sample. From the 3-D image of the Cl^‒^ ion map, the localisation of the ions within the attack site is verified. Regarding the remaining analysed volume, it is observed that most of the chlorides are only present on the top-most surface, particularly near IMPs, and the intensity reduces as we go deeper into the substrate.

From the Cu^‒^ and CuO^‒^ images, the sites of the IMPs of the alloy are identified both within and outside the attack site. This point has two major implications for this work. The first is that not all the IMPs were attacked during the 24 h immersion in a 2-MBT-containing NaCl solution, indicating protection by the inhibitor. The second is that the attack site formed here is around the site of a cluster of particles, as obvious from the 3-D display of the Cu^‒^ map. This suggests that the attack site is most likely a combination of trenching around the particles and dealloying of the particles themselves, resulting in what appears to be a large pit. This observation is in agreement with the mechanisms of IMP attack and dealloying from literature^55^.

To understand the effects of 2-MBT on the dealloying process for the IMPs, we examine the MgO^‒^, MnO^‒^, FeO^‒^, and Si^‒^ ions maps. While MgO^‒^ is observed rather homogeneously within the analysed volume, it is also found locally within the attack region (pit) at two distinct sites, and outside the attack region at one site. However, these sites do not exhibit particularly higher intensities compared to the intensity of the MgO^‒^ within the matrix, suggesting that the Al-Cu-Mg (S phase) particles are most likely attacked to a large degree. Considering the MnO^‒^, FeO^‒^, and Si^‒^ ions chemical maps, while the IMPs are distinctly observed outside the attack site, they are less evident within the pit. This suggests that the Al-Cu-Fe-Mn-(Si) particles outside the attack site are rather well protected from corrosion attack after exposure to the 2-MBT containing NaCl solution. However, this is not the case within the pit, where the IMPs are attacked to a greater degree indicating partial dealloying, as evidenced by the very low intensity of these ions within the pit.

The CSN^‒^, CN^‒^, and C_2_S^‒^ ion maps, which correspond to various fragments of the 2-MBT molecule^24–26^, illustrate a clear trend for the localisation of these ions at the sites of the IMPs. This is also observed clearly in the 3D images of these ions. The highest intensity for these species is found at the centre of the attack site, exhibiting good correlation with the ions corresponding to the IMPs. Additionally, the IMP sites corresponding to the Al-Cu-Mg (S phase), Al-Cu-Mn-Fe-(Si) phase, and Al-Cu (θ phase) particles outside the attack site also show a high intensity of these ions, although slightly lower than within the pit. These observations irrefutably show that 2-MBT is adsorbed on the intermetallic particles, both on the surface and within the pits. Finally, from the 3D images, a relatively high intensity of the 2-MBT corresponding ions is observed near the top-most surface which decreases when going deeper into the substrate. This observation is also consistent with the occurrence of a thin 2-MBT layer on the top-most surface.

### Adsorption & Inhibition Mechanisms of 2-MBT on AA2024 T3

**Fig. 20:**
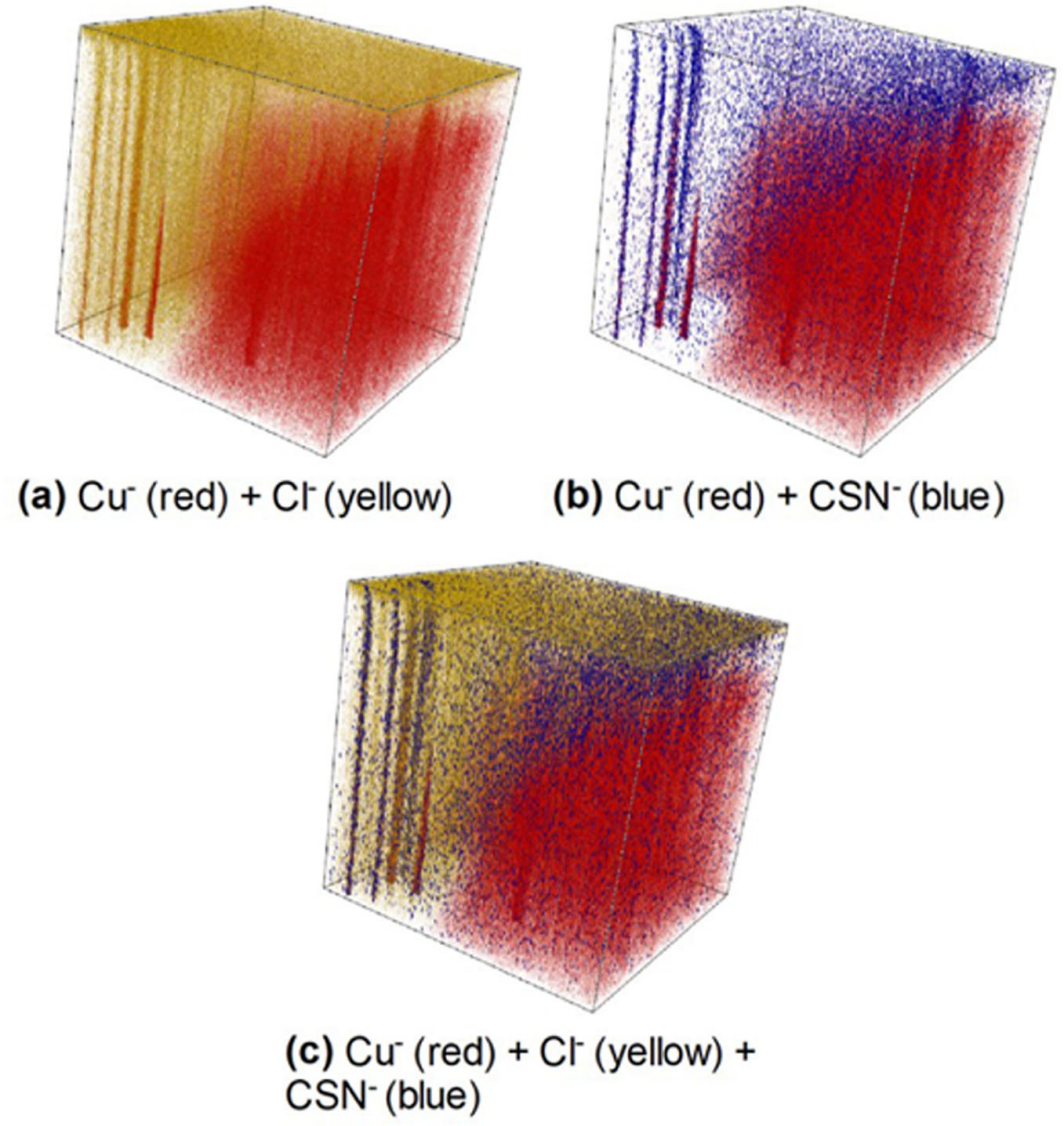
Combined plots of ToF-SIMS 3-D imaging revealing the mechanisms of 2-MBT inhibitor adsorption and action. (**a**) ^Cu‒^+^Cl‒^ overlay, (**b**) ^Cu‒^+^CSN‒^ overlay, and (**c**) ^Cu‒^+^Cl‒^+^CSN‒^ overlay. The analysed depth is 1000 s of sputtering time with the top face corresponding to 0 seconds of sputtering time, and the total area analysed is 160 × 160 μm².

While surface analysis by XPS (Fig. 15) and ToF-SIMS depth profiling (Figs. 16 and 17) clearly revealed the presence of 2-MBT on the sample surface, it was not sufficient to fully understand the adsorption and inhibition mechanisms. On the other hand, ToF-SIMS 3-D imaging provides us with crucial information to further comprehend the system. Combining the 3-D images of the Cl^‒^ and Cu^‒^ ions in a common 3-D plot (Fig. 20a), it is observed that the chloride ions are essential to expose the intermetallic particles by attack through the oxide film. From the chemical maps of the sample immersed in the reference NaCl solution, Fig. 13, it is observed that pits and trenches are formed at the sites of intermetallic particles due to the attack by the chloride ions. This feature, also observed in Fig. 18 for the sample immersed in 2-MBT-containing NaCl solution, exposes the IMPs to the 2-MBT inhibitor dissolved in the solution.

Next, the combination of the Cu^‒^ and CSN^‒^ ion 3-D images (Fig. 20b) shows that the inhibitor is located directly on top of the copper-rich IMPs. Additionally, they are also found on the top-most surface and the interface between the oxide region and the alloy substrate. This confirms our hypothesis that the molecule adsorbs mostly on the intermetallic particles but also on the alloy matrix. However, adsorption of 2-MBT on the IMPs would not have been possible without the presence of Cl^‒^ ions which break down the native oxide film and thus expose the IMPs to the inhibitor. Compiling the three ions 3-D images, we obtain Fig. 20c, where the role of chloride ions and the adsorption of 2-MBT on the intermetallic particles of the AA2024 T3 alloy are illustrated.

**Fig. 21:**
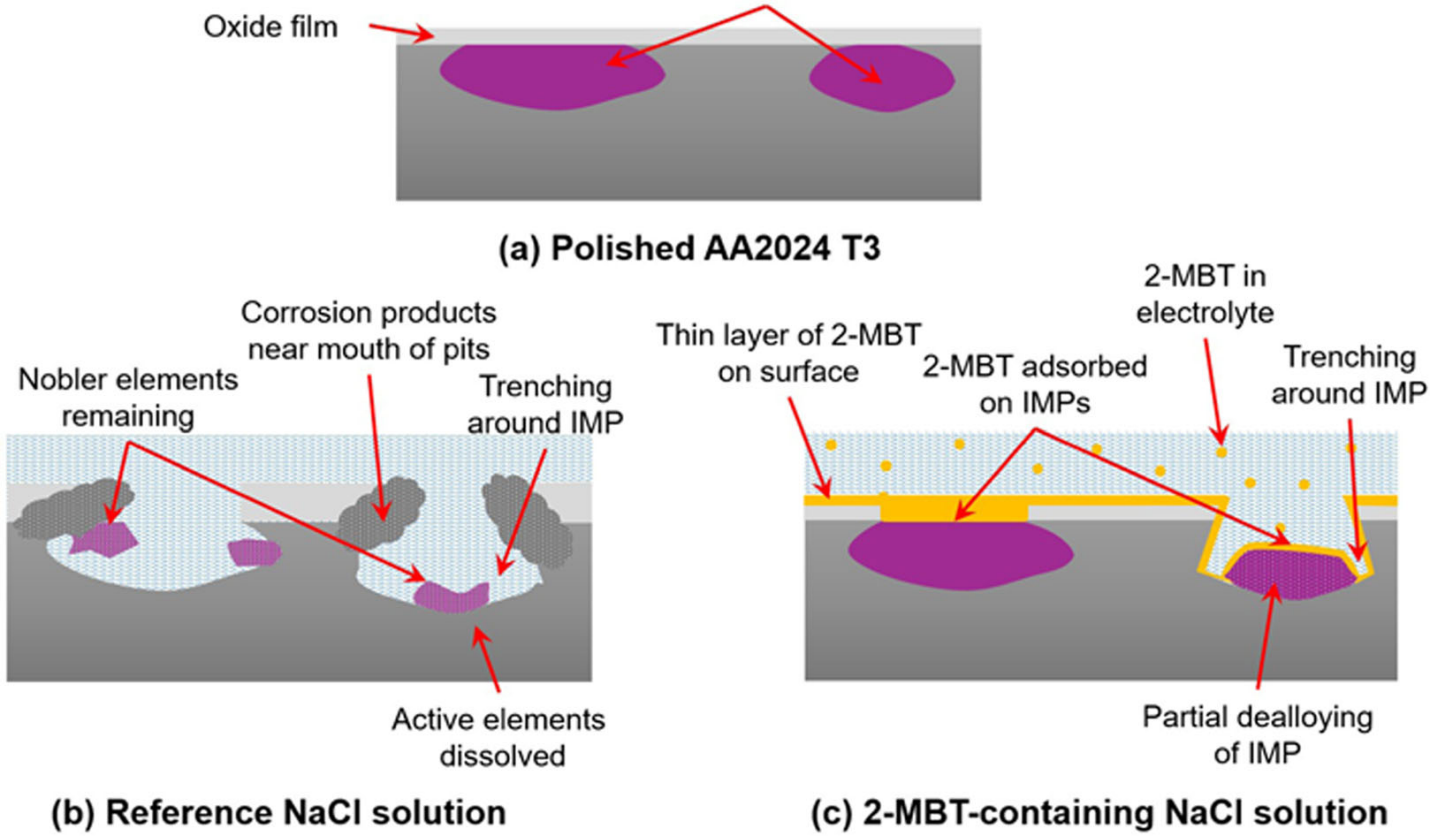
Scheme illustrating the mechanisms of corrosion attack and inhibitor action on a model of the AA2024 T3 alloy. (**a**) Polished sample with intermetallic particles, (**b**) Mechanism of corrosion attack and dealloying of intermetallic particles in a reference NaCl solution, (**c**) 2-MBT adsorption and inhibition mechanisms at intermetallic particle sites and the alloy surface in a 2-MBT-containing NaCl solution.

Based on the results obtained in this work, a scheme is proposed in Fig. 21 for the adsorption of 2-MBT inhibitor on the AA2024 T3 alloy in a neutral chloride solution, along with the mechanism of intermetallic particle dealloying in presence of chloride ions. Figure 21a shows a model alloy sample with intermetallic particles exposed at the surface by mechanical polishing, later covered by an oxide film due to oxidation of the sample in air. The dealloying process that occurs on the intermetallic particles is shown in Fig. 21b, where the more active elements such as Mg and Al in the Al_2_CuMg phase particles and the Al and Mn in the Al-Cu-Mn-Fe-(Si) particles are attacked, forming corrosion products that are deposited at the mouth of the pits. This dealloying process leaves behind the nobler elements from the IMPs such as Cu and Fe within the pits, as evidenced from the Cu^‒^ ion chemical map in Fig. 13, or redeposited around the pits^12–14^.

Finally, in Fig. 21c, the mechanism of inhibitor adsorption and action derived from the results of this work is shown. From the chemical mapping images and the 3-D images (Figs. 18 and 19), it is concluded that not all the IMPs on the alloy substrate were severely attacked during 24 h immersion in a 2-MBT-containing NaCl solution. Most of the IMPs were exposed to the inhibitor by the penetration of the chloride ions through the oxide film, thus resulting in 2-MBT molecule adsorption on the mildly attacked IMPs.

On the other hand, the particles observed inside the attack site demonstrated a different adsorption mechanism. These particles, exposed by Cl-induced trenching of the alloy matrix around them, were also attacked by the chloride ions to initiate dealloying, as evidenced in Fig. 20. However, these particles only exhibited partial dealloying, as indicated by the presence of low intensities of MgO^‒^ and MnO^‒^ ions within the attack site in Figs. 18 and 19. Nevertheless, the partial dealloying process results in a higher Cu concentration within the particle, thereby promoting 2-MBT inhibitor adsorption more effectively, as indicated by the relatively higher intensity of the molecule fragments on these sites in Figs. 18 and 19.

A thin layer of 2-MBT was also adsorbed on the surface of the sample at the alloy substrate/oxide interface, as evidenced in Figs. 16 and 17. While this 2-MBT layer restricts the growth of the oxide film, it is not defect-free, thereby resulting in oxide growth at insufficiently protected regions (within the attack sites and pits) after exposure to air. The fact that the quantity of adsorbed 2-MBT was rather low on the surface, as determined by the N 1s and S 2p spectra, would explain why 2-MBT demonstrates a reversible behaviour as indicated by Visser et al.^32^. This is why a sufficiently high concentration of the inhibitor is required to maintain optimal protection, as the molecules would be consumed upon further corrosion attack. Nevertheless, the combination of adsorbed 2-MBT on the IMPs and the 2-MBT layer on the alloy matrix results in inhibition of corrosion attack and inhibition of oxide growth on the alloy.

In summary, it is established that 2-MBT effectively inhibits corrosion of the AA2024 T3 alloy in neutral chloride media, increasing the polarisation resistance, shifting the corrosion potential to a nobler value, and lowering the corrosion current density. 2-MBT adsorption is evidenced from the S 2p and N 1s core level spectra from XPS analysis. A thin 2-MBT layer is formed on the top-most surface of the alloy and at the oxide/substrate interface near attack sites, thereby protecting the alloy matrix from corrosion attack and surface oxidation. It is also determined that 2-MBT adsorbs on intermetallic particles emerging at the surface, as shown by ToF-SIMS 3-D chemical mapping. The chloride ions breakdown the native oxide film at the IMP sites and initiate the dealloying process of the IMPs. This allows the 2-MBT molecules to adsorb on the partially dealloyed Cu-rich particles and thereby inhibit further corrosion attack.

## Methods

### Material and sample preparation

The samples used for this work were commercially produced AA2024 T3 aluminium alloy samples, obtained from the Institute of Research and Technology – Materials, Metallurgy, and Processes (IRT-M2P), France. The chemical composition of the alloy is given in Table 2. The alloy is designated with the T3 temper indicating that it was solution heat treated followed by cold working and naturally aged to a substantially stable condition^1^. This treatment is applied to improve strength and to stabilise the mechanical properties of the alloy.

The samples were cut into square coupons of 1 × 1 cm for experimental analysis. The samples were first mechanically abraded using silicon carbide papers from 800 grit down to 4000 grit using ultra-pure water (Millipore Technologies, resistivity > 18.2 MΩ.cm) as lubricant. This was followed by mechanical polishing using diamond paste from 6 μm down to 0.25 μm to obtain a mirror-finish surface. Finally, the samples were cleaned ultrasonically in successive baths of acetone, ethanol, and ultra-pure water for 3 min each, followed by drying using a stream of pure nitrogen gas.

### Electrochemical measurements

The experiments were performed using two separate electrolytes – the reference solution (no inhibitor), and the inhibitor-containing solution. The reference solution was a 0.01 M NaCl aqueous solution of near-neutral pH in aerated conditions at room temperature. A low concentration of NaCl was used to limit aggressiveness of the electrolyte and reduce the reaction rate which prevents the formation of a very thick corrosion product layer on the surface, thereby allowing us to study the initial corrosion mechanisms using a surface science approach. The inhibitor-containing solution used was a 0.01 M NaCl solution with dissolved 0.1 mM 2-MBT. Since the solubility of 2-MBT is very low at neutral pH^56^, the 2-MBT powder was dissolved in the NaCl solution by heating at 70 °C for a period of 10 days with constant stirring. Once dissolved, the solution was topped off with ultra-pure water to compensate for evaporation losses, thus maintaining the concentration of 2-MBT and NaCl. The solutions were prepared using ultra-pure water and analytical grade chemicals (Sigma Aldrich).

The electrochemical experiments were conducted in a classical three-electrode electrochemical cell using a Biologic SP 200 potentiostat and EC-lab collection software. The sample was used as working electrode, a platinum plate mesh as counter electrode, and a saturated calomel electrode (SCE) as reference electrode (+0.2415 V vs SHE). The working electrode area, delimited by an O-ring, was 0.16 cm². OCP and LPR of the system were monitored over 24 h of experimental time. OCP was monitored for 1 h intervals followed by LPR measurements over ±10 mV versus OCP using a sweep rate of 0.167 mV/s (total scan time of 2 min) at each 1 h interval. A linear fit was applied to the LPR measurements to obtain the polarisation resistance (*R*_*p*_) of the sample as a function of time. This was determined by taking the inverse of the slope of the current density vs potential curve at the free corrosion potential. The PDP tests were performed using linear sweep voltammetry after keeping the samples at OCP conditions for 24 h. The samples were polarised from –0.25 to +0.50 V vs OCP, with a sweep rate of 1 mV/s, similar to the work of Visser et al.^32^. The corrosion potential (*E*_corr_) of the samples was determined by identifying the potential of the sample at zero current during the PDP measurements. The electrochemical experiments were repeated threefold to ensure reproducibility of the results.

Optical microscopy (OM) was performed on the samples after 24 h immersion in the electrolytes to observe the corrosion morphology. Once removed from the electrochemical cell, the samples were rinsed with ultra-pure water and dried in a stream of pure nitrogen gas. A digital Keyence VHX 5000 microscope was used for imaging and post-imaging analysis was performed using the Keyence software.

### X-ray photoelectron spectroscopy (XPS)

Surface analysis was performed on the AA2024 T3 samples after (i) mechanical polishing, (ii) immersion in the reference solution (no inhibitor) for 24 h in OCP conditions, and (iii) immersion in the 2-MBT-containing solution (with inhibitor) for 24 h in OCP conditions. Once disconnected from the electrochemical cell, the samples were rinsed using ultra-pure water and dried in a stream of nitrogen gas. They were then transferred to the ultra-high vacuum (UHV) chamber of the spectrometers for analysis. XPS analysis was performed using a Thermo Electron ESCALAB 250 Xi spectrometer with a monochromated Al Kα X-ray source (hv = 1486.6 eV). The base pressure of the system was less than 10^‒10^ mbar, and the analysis was performed using an X-ray spot size of 900 μm in diameter at the centre of the area that was exposed to the electrolyte. Survey spectra were recorded with a pass energy of 100 eV at a step size of 1 eV. High resolution spectra of the Al 2p, C 1s, Cl 2p, Cu 2p, Fe 2p, Mg 2p, Mn 2p, N 1s, O 1s, S 2p, Si 2p, Ti 2p, Zn 2p, Zr 3d core levels, Fermi level, and Cu LMM Auger transition were recorded with a pass energy of 20 eV at a step size of 0.1 eV and at 90° take-off angle. The Fermi level was used as a reference for all binding energies (BEs). Curve fitting of the spectra was performed with the CasaXPS software using an adjustable Shirley background. The photoionization cross-section values at 1486.6 eV were taken from the Scofield database^57^, the transmission function of the analyser was given by Thermo Fischer, and the inelastic mean free paths were determined using the TPP-2M formula^58^.

### Time-of-flight secondary ion mass spectrometry (ToF-SIMS)

ToF-SIMS analysis was performed using an IonTof ToF-SIMS 5 spectrometer with a base pressure below 5 × 10^‒9^ mbar. Data acquisition and post processing analysis were carried out using the SurfaceLab software version 7.2. Bismuth (Bi^+^) primary ions of 25 keV energy at a target current of 1.2 pA were used in a high current (HC) bunched mode for performing analysis over an area of 100 × 100 μm². The area of analysis was selected by choosing the region with the largest homogeneity of attack type visible on the surface. The images of the analysed areas are given in Figs. S2 and S3 in the supplementary information. Depth profiles were obtained by interlacing analysis in these static SIMS conditions with sputtering. Sputtering was performed using a Caesium (Cs^+^) ion gun of 1 keV energy delivering a 35 nA target current over an area of 500 × 500 μm². For depth profiling limited to the extreme surface, the sputtering parameters were marked down to 0.5 keV energy of Cs^+^ ions delivering a 20 nA target current over a similar area to obtain a better time resolution for the initial sputtering seconds. 3-D chemical imaging was performed in Burst Alignment (BA) image mode (Bi^+^ ions, 25 keV, 0.3 pA, 200 × 200 μm²) using similar sputtering parameters (Cs^+^ ions, 1 keV, 35 nA, 500 × 500 μm²) as those of the depth profiles. Both ion beams were aligned at an incidence of 45° with respect to the sample surface to ensure analysis at the centre of the sputtered crater. The acquisitions were performed in negative polarity, thereby allowing us to analyse both the organic regions and the oxide (inorganic) regions of the surface. To calibrate the data acquired, the exact mass values of at least seven known species with low and high masses were utilised, thereby correcting any error with the mass spectrum. Due to the differential sputtering rates of different layers by ToF-SIMS, the analysed depth is left as sputtering time (seconds).

## Supplementary information

Supplementary Information

## Data Availability

Data will be made available from the corresponding author on request.
