# Supplementary material for: Corrosion inhibition mechanisms of 2-mercaptobenzothiazole on AA2024 T3 aluminium alloy
Source: Npj Mater Degrad. 2025 Aug 3;9(1):100. doi: 10.1038/s41529-025-00653-z (PMC12317847; doi:10.1038/s41529-025-00653-z)
Supplement: Supplementary file 1 — Supplementary Information [file 41529_2025_653_MOESM1_ESM.docx]

*Supplementary Information to:*

**Corrosion inhibition mechanisms of
2-mercaptobenzothiazole on AA2024 T3 aluminium alloy**

Vishant Garg^†,^ *, Maxime Magnan, Sandrine Zanna, Antoine Seyeux,
Frédéric Wiame, Vincent Maurice, Philippe Marcus*

^1^ *PSL University, CNRS – Chimie ParisTech, Institut de Recherche de Chimie Paris, Physical Chemistry of Surfaces Research Group,
11 rue Pierre et Marie Curie, 75005 Paris, France*

^†^ Present address: *Institute of Research and Technology – Materials, Metallurgy, and Processes (IRT-M2P), 12 rue de l’Artisanat, 67120 Duppigheim, France*

* Corresponding authors for the work:

[vishant.garg@chimieparistech.psl.eu](mailto:vishant.garg@chimieparistech.psl.eu)

[philippe.marcus@chimieparistech.psl.eu](mailto:philippe.marcus@chimieparistech.psl.eu)


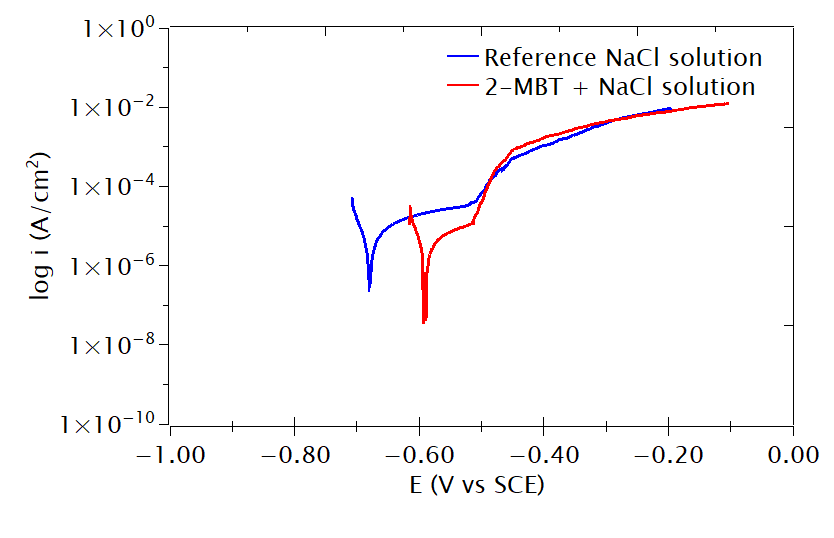


*Figure S1: Anodic polarisation curves of the AA2024 T3 samples obtained after 24 hours immersion in the reference NaCl solution and the 2-MBT-containing NaCl solution. The scans were performed from -0.05 V to +0.50 V vs OCP using a scan rate of 1 mV/s.*


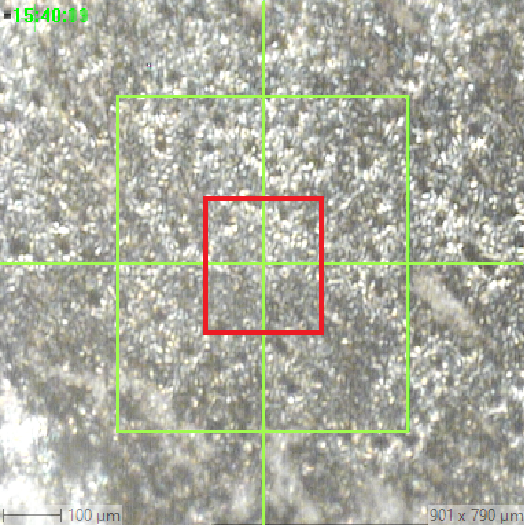


*Figure S2: Image showing the area analysed by ToF-SIMS on the AA2024 alloy sample immersed in the NaCl reference solution. The green box represents the area that was sputtered during the ToF-SIMS measurements while the red box represents the area that was analysed to obtain the 3D chemical maps and the depth profiles presented in Figures 12, 13, and 14.*


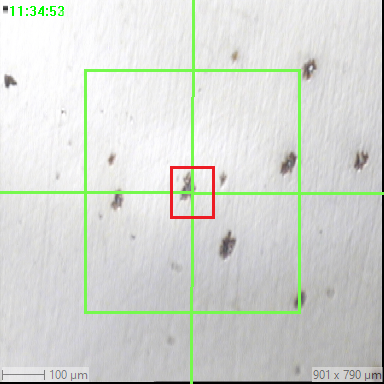


*Figure S3: Image showing the area analysed by ToF-SIMS on the AA2024 alloy sample immersed in the 2-MBT containing NaCl solution. The green box represents the area that was sputtered during the ToF-SIMS measurements while the red box represents the area that was analysed to obtain the 3D chemical maps and the depth profiles presented in Figures 16, 17, 18, and 19.*
